# The Relationship Between Work–Family Conflict and Health Behaviors: A Systematic Review and Meta-Analysis

**DOI:** 10.3390/bs16030386

**Published:** 2026-03-07

**Authors:** Xiyu Peng, Ze Chen, Yu Li, Shuai Yuan, Jieling Chen

**Affiliations:** 1 School of Nursing, Sun Yat-sen University, Guangzhou 510080, China; pengxy58@mail2.sysu.edu.cn (X.P.); liyu253@mail2.sysu.edu.cn (Y.L.); 2 School of Humanities and Social Science, The Chinese University of Hong Kong, Shenzhen 518172, China; zechen1@link.cuhk.edu.cn; 3 School of Business, University of Amsterdam, 1012 VT Amsterdam, The Netherlands; s.yuan@uva.nl

**Keywords:** meta-analysis, health behaviors, work–family conflict

## Abstract

As family structures and workforce compositions evolve, individuals increasingly navigate multiple roles across work and family domains. Despite growing research interest, a comprehensive synthesis examining the relationship between work–family conflict and health behaviors remains absent. This systematic review and meta-analysis addresses this significant gap by quantifying associations between work–family conflict and five important health behaviors: sleep disturbances, smoking behaviors, alcohol consumption, physical activity levels, and healthy diet. The Preferred Reporting Items for Systematic Reviews and Meta-Analyses (PRISMA) guidelines were followed. Electronic databases (PubMed, Embase, PsycINFO, Web of Science) were searched. Heterogeneity and publication bias were assessed using forest plots, *I*^2^, Cochran’s Q-statistics, Funnel plots, and the Egger test, respectively. A total of 33 articles were included in this systematic review and meta-analysis. Work–family conflict had a small-to-medium positive association with sleep disturbances (*r* = 0.188; 95% CI [0.128, 0.247]); a negative association with healthy diet (*r* = −0.129; 95% CI [−0.219, −0.037]); and a modest and positive association with smoking behaviors (*r* = 0.082; 95% CI [0.033, 0.206]) and alcohol consumption (*r* = 0.074; 95% CI [0.039, 0.109]). The findings of this study suggest that individual, family, and organizational strategies reducing work–family conflict may facilitate the development and adoption of healthier behaviors, such as improving sleep and dietary practices. This study enhances understanding of work–family conflict’s relationship with health behaviors, bridging the management and occupational health psychology literature with the general and public health literature by systematically examining, for the first time, how work–family conflicts impair personal health behaviors.

## 1. Introduction

Contemporary changes in family structure and labor force composition increasingly require individuals to fulfill multiple work and family roles at the same time. Meanwhile, rapid digital transformation and the widespread adoption of remote and hybrid work models have further blurred the boundaries between work tasks and family responsibilities (Allen et al., 2021).Work–family conflict represents a type of stress arising when work and family roles prove incompatible, impeding an individual’s effective functioning in both roles (Greenhaus & Beutell, 1985). This conflict contributes to a wide range of health problems, including mental disorders (Amstad et al., 2011), burnout (Zhou et al., 2024), cardiometabolic disorders (Saiki et al., 2024), musculoskeletal pain (Weale et al., 2021), and somatic symptoms (Amstad et al., 2011). Given the negative consequences of work–family conflicts on multiple aspects of one’s work and personal life, the study of work–family interfaces and conflicts has emerged as one of the most foundational and prominent research domains within occupational health psychology. However, research in occupational health psychology and management has traditionally focused on performance outcomes or psychological consequences of work–family conflict, while largely overlooking health-related behavioral outcomes. This study therefore aims to supplement existing occupational health psychology and general health literature by systematically reviewing the consequences of work–family conflict on health behaviors that extend beyond the work domain.

Several theoretical perspectives explain the link between work–family conflict and health behaviors. Stress theory posits that individuals employ unhealthy behaviors as emotional management tools, seeking pleasure to mitigate stress (Ng & Jeffery, 2003). Work–family conflict serves as a stressor, triggering stress-induced responses that, in turn, drive unhealthy behavioral choices, including excessive alcohol consumption (Scholarios et al., 2017), smoking (Nelson et al., 2012), and overeating high-fat foods (Shukri et al., 2018). These behaviors may also serve as maladaptive coping mechanisms to deal with the stress and strain caused by work–family conflict. Conservation of Resources (COR) theory (Hobfoll, 2002) provides another explanation, recognizing time and energy as finite resources. Work and family compete for these resources, with allocation to one domain reducing availability for others. Individuals experiencing work–family conflict often lack time for healthy activities like exercise (Dias et al., 2017), healthy meal preparation (Shukri et al., 2018), or adequate sleep (Magee et al., 2018). This conflict continuously depletes energy, diminishing motivation for healthy behaviors while simultaneously increasing susceptibility to unhealthy coping behaviors. It is important to note that work–family conflict is bidirectional: work-to-family conflict occurs when work demands interfere with family life, whereas family-to-work conflict occurs when family demands interfere with work performance (Netemeyer et al., 1996). These two directions have distinct antecedents and consequences. Family-to-work conflict is more strongly associated with work-related stressors and job outcomes, while family-to-work conflict is more closely linked to family-related stressors and personal outcomes (Amstad et al., 2011). However, due to the limited number of studies reporting effect sizes separately for work–family conflict and family-to-work conflict in relation to health behaviors, this meta-analysis primarily focuses on overall work–family conflict. Nonetheless, we acknowledge this distinction as an important direction for future research.

Currently, reviews of work–family conflict have examined antecedents such as personality traits and job characteristics (Miller et al., 2022), and outcomes spanning across work-related factors (e.g., turnover intention, job satisfaction, sick leave) (Ford et al., 2007; Nilsen et al., 2017; Yildiz et al., 2021), and well-being and mental health outcomes (e.g., insomnia, and anxiety and depression) (Miller et al., 2022; Yang et al., 2018). However, there remains a notable gap in the literature regarding a comprehensive review that systematically examines work–family conflict’s relationship with various health behaviors. Consequently, the magnitude and variation in this association across different health behaviors remain almost entirely undetermined. For example, work–family conflict was found to be negatively related to physical activity among mixed-occupation employees (Wei et al., 2023), but showed no significant association in navy populations (Guérin & Gottschall, 2024). These inconsistent findings necessitate further investigation; the current systematic review and meta-analysis can potentially provide much-anticipated clarity by synthesizing disparate results and quantifying overall effect sizes across studies conducted in different contexts and situations. Moreover, the experience and consequences of work–family conflict may not be uniform across genders. Women continue to bear a disproportionate share of domestic labor and childcare responsibilities in most societies, which may render them more vulnerable to the negative effects of work–family conflict on health and well-being (Taouk et al., 2025). However, the extent to which gender moderates the relationship between work–family conflict and specific health behaviors remains underexplored. This review therefore also considers, where data permit, the potential role of gender in shaping these associations.

This systematic review and meta-analysis quantifies associations between work–family conflict and five health behaviors (sleep disturbances, smoking behaviors, alcohol consumption, physical activity levels, healthy diet). Of note is that sleep represents a crucial healthy lifestyle component (Kaminsky et al., 2022). To better align with our overarching objective of assessing how work–family conflicts affect heath behaviors, we focused on sleep disturbances, an indicator of sleep hygiene behaviors.

Theoretically, this study advances the literature by moving beyond the replication of established stress and resource theories. Rather than merely testing whether work–family conflict relates to health behaviors, we systematically compare the magnitude of these associations across five behavioral domains. This comparative approach allows us to identify which health behaviors are more or less susceptible to work–family conflict, thereby offering a nuanced theoretical insight: Health behaviors differ in their sensitivity to resource depletion and emotional distress. By mapping these differential effects, this study provides an empirical foundation for developing behavior-specific intervention strategies—a contribution that extends prior meta-analyses focused on singular outcomes or undifferentiated health aggregates.

## 2. Methods

### 2.1. Literature Search

We conducted this systematic review and meta-analysis following the PICO-SD (Population, Intervention, Comparator, Outcome, Study Design) framework and PRISMA (Preferred Reporting Items for Systematic Reviews and Meta-Analyses) guidelines (Table S1) (Page et al., 2021). We searched Web of Science, Embase, PubMed, Embase and PsycINFO to identify research papers published through 4 March 2025. To ensure comprehensiveness, the search was conducted without a start date, covering all the literature since the database’s inception. We recognize that this may result in the inclusion of some early, non-relevant literature in the search results; however, this was filtered through rigorous inclusion/exclusion criteria. Search keywords combined MeSH terms and entry terms for comprehensive coverage. We performed additional manual searches reviewing bibliographies of included papers. Table S2 presents the detailed search strategy.

### 2.2. Eligible Criteria and Study Selection

The retrieved studies were selected if they: (1) examined work–family conflict; (2) evaluated at least one type of health behavior outcome; (3) reported quantitative associations between work–family conflict and health behaviors; and (4) utilized an observational design.

Studies were excluded if they (1) were qualitative studies, intervention studies, reviews (including narrative and conceptual reviews), systematic reviews and meta-analyses, research protocols, and editorials; (2) were not published in English; or (3) were not available in full text.

The literature search and selection process was conducted independently by 2 evaluators. Any inconsistencies between reviewers during the literature selection process were resolved by discussion with the research team to determine the eligibility of the studies. When multiple studies examined identical participant groups, we considered them duplicates and selected one combined study for analysis.

### 2.3. Data Extraction and Risk of Bias Assessment

The following data were extracted from the included studies using standardized tables: (1) characteristics of the study (first author, year of publication, study country/region, study design); (2) characteristics of the sample (sample size, mean age, sex ratio, occupation, role in family); (3) measurement method of work–family conflict; (4) type of health behaviors and its measurement method; and (5) type of effect indicator for the association between work–family conflict and health behaviors, and its effect size. Any inconsistencies or ambiguities were resolved through discussion with the research team.

We evaluated the quality of studies using the criteria proposed by Kmet et al. (2004), a comprehensive assessment tool for evaluating quantitative studies across 14 items, including study design, sample selection, sample characteristics, measurement tools, statistical methods, presentation of results, and consideration of confounders. Each criterion received ratings on a 4-point scale (yes = 2, partial = l, no = 0, NA). Summary scores exceeding 65% of the maximal score indicated moderate-to-high quality. Two investigators independently assessed study quality. The disagreements were again resolved by discussion to reach consensus. Quality assessment using Kmet et al. (2004) criteria indicated that all 33 studies had a low risk of bias, with summary scores above 65%. Detailed scores are provided in Table S4.

### 2.4. Synthesis of the Results

In this manuscript, we follow the conventional notation in meta-analysis: *k* refers to the number of studies contributing to a given analysis, and *n* refers to the total number of participants across those studies. This notation is used consistently throughout the Results section and tables.

To qualitatively analyze the results, we first provided an overview of the study characteristics, sample characteristics and assessment of the work–family conflict and health behaviors. As noted, five types of health behaviors include sleep disturbances, alcohol consumption, smoking behaviors, physical activity levels, and healthy diet.

For quantitative synthesis, we estimated pooled correlation coefficients using Fisher’s z-transform and inverse transformation (J. P. T. Higgins, 2008). Cochran’s Q-statistics and *I*^2^-statistics were used to assess the heterogeneity of the studies included in the meta-analysis. Meta-analysis is the statistical combination of results from two or more separate studies (J. Higgins et al., 2024). Given that this study aims to explore the association, we conducted meta-analysis to synthesize findings from at least three studies. Given that significant heterogeneity between effect sizes was detected (Q-statistics *p* < 0.10 and *I*^2^ ≥ 50%) (J. P. T. Higgins, 2008), we employed random-effects models. When articles reported multiple types of health behaviors, each represented a separate effect size. When an individual study reported effect sizes for work–family conflict in two directions (e.g., work interference with family, family interference with work) separately or in different dimensions (e.g., time-based, strain-based), we calculated the weighted average effect sizes to represent the overall effect size for this study. The confidence interval (CI) for the pooled correlation was calculated and a 95% CI excluding zero indicated statistical significance. The magnitude of the pooled correlation coefficients was evaluated using the Updated Cohen’s criteria (Gignac & Szodorai, 2016), 0.10 (small), 0.20 (moderate), 0.30 (large). To test the validity of the findings, publication bias was assessed using funnel plots and Egger regression. If the funnel plot is significantly asymmetric and the significance of the Egger regression is less than 0.05, publication bias is present. When publication bias was suspected, we used the trim-and-fill methods to estimate the extent to which publication bias affected the validity of the findings. All statistical analyses were performed using Comprehensive Meta-analysis, version 3 software (Biostat).

## 3. Results

### 3.1. Study Selection

**Figure 1:**
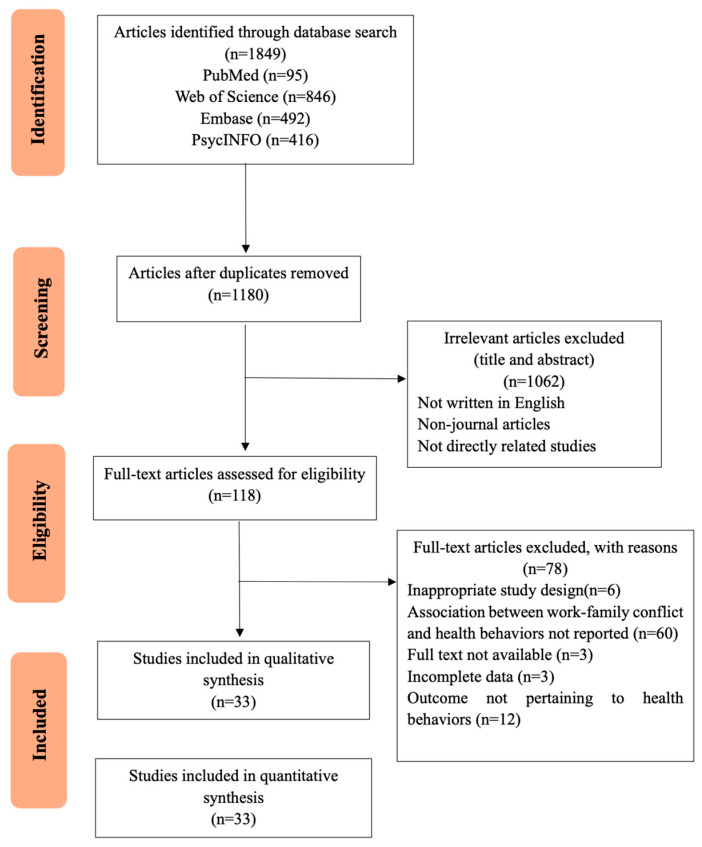
The PRISMA (Preferred Reporting Items for Systematic Reviews and Meta-Analyses) flowchart of the study selection process.

Of 1180 non-duplicate studies identified, 1062 were excluded after reviewing titles and abstracts. The remaining 118 studies were assessed for eligibility through a full-text review. Ultimately, 33 studies met the inclusion criteria of our systematic review, providing 39 effect sizes. Some articles simultaneously provide effect sizes for work-family conflict in relation to sleep disturbances, smoking behaviors, alcohol consumption, physical activity levels, and other factors. Details of the study selection process and reasons for exclusion are displayed in Figure 1. Figure 1 presents the PRISMA flowchart. Of the 1180 non-duplicate studies identified, 1062 were excluded after title and abstract screening. The remaining 118 studies were assessed in full text, and 33 studies met the inclusion criteria.

### 3.2. Characteristics of the Reviewed Studies

The overall characteristics of the included studies are summarized in Table S3. Of the 33 studies, most employed cross-sectional designs (*k* = 23), with fewer longitudinal studies (*k* = 10). The age distributions of participants varied, with younger samples (mean age < 40; *k* = 11), older samples (mean age > 40; *k* = 14), and balanced distributions (wide age range; *k* = 4). Gender distributions included female majority samples (≥75% female; *k* = 4), male majority samples (≤25% female; *k* = 5), and balanced samples (>25% female and <75% female; *k* = 23). All study participants were employed in a diverse range of occupations, with specific studies examining nurses, drivers, teachers, police, navy, and IT workers. The majority of studies reported that most participants were married and/or had children living in the household, which were used as proxies for family caregiving responsibilities. However, it should be noted that marital status and parenthood do not uniformly translate into caregiving burden, as the intensity of domestic and childcare responsibilities varies considerably across individuals and cultural contexts.

### 3.3. Risk of Bias Assessment

The risk of bias assessment showed that all 33 studies had a low risk of bias. Inter-rater reliability showed strong agreement (Kappa = 0.82; 95% CI [0.48, 1.00]), indicating robust assessment reliability. The individual scores for each study are shown in Table S4.

### 3.4. Measurement of Work–Family Conflict and Health Behaviors

The reviewed studies examined five types of health behaviors, including sleep disturbances (*k* = 11), alcohol consumption (*k* = 11), smoking behaviors (*k* = 5), physical activity levels (*k* = 8), and healthy diet (*k* = 3) (Table S5).

In the 33 studies, work–family conflict was measured by a variety of instruments. The eight-item scale by Netemeyer et al. (1996) and multidimensional scale by Carlson et al. (2000) were most frequently employed. Some studies used self-developed items.

Regarding the health behaviors, sleep disturbances was primarily measured by the Pittsburgh Sleep Index Scale (Buysse et al., 1989), Basic Nordic Sleep Questionnaire (Partinen & Gislason, 1995), and the Jenkins Sleep Questionnaire (Jenkins et al., 1988). Physical activity levels was primarily measured by the Leisure Time Exercise Questionnaire (Godin, 2011), the International Physical Activity Questionnaire, and self-developed items. Alcohol consumption was measured by self-reported single-item items reflecting the amount and frequency of alcohol use, as well as by scales such as the CAGE (Mayfield et al., 1974) and the Alcohol Use Disorders Identification Test (AUDIT) (Osaki et al., 2014), which reflect alcohol dependence and problems. Smoking behaviors was primarily measured by self-developed single-entry measures of whether and how much tobacco is smoked. Healthy diet was measured by the Adapted Healthy Eating Index (AHEI) (Norte Navarro & Ortiz Moncada, 2011), food frequency screener module (Moore et al., 2015) and self-developed items.

### 3.5. The Relationship Between Work–Life Conflict and Health Behaviors

**Figure 2:**
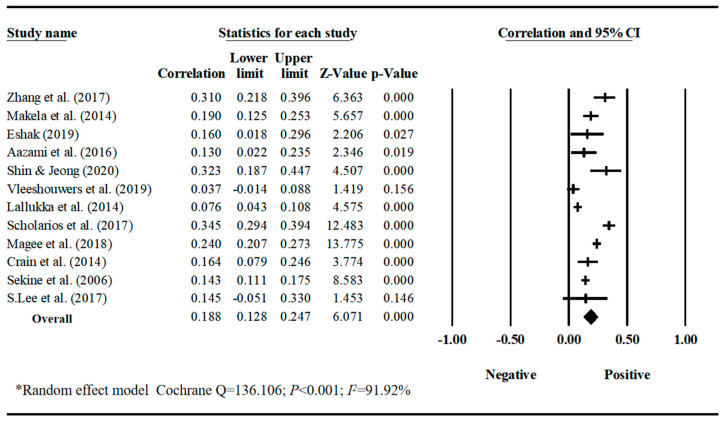
Forest plot of the correlation coefficients between work–family conflict and sleep disturbances. Note: Black squares/crosses indicate study-specific correlation coefficients, horizontal lines represent 95% confidence intervals, and the black diamond denotes the overall pooled effect size estimated using a random-effects model. Data sourced from the following studies: (Zhang et al., 2017; Makela et al., 2014; Eshak, 2019; Aazami et al., 2016; Shin & Jeong, 2020; Vleeshouwers et al., 2019; Lallukka et al., 2014; Scholarios et al., 2017; Magee et al., 2018; Crain et al., 2014; Sekine et al., 2006; S. Lee et al., 2017).

**Figure 3:**
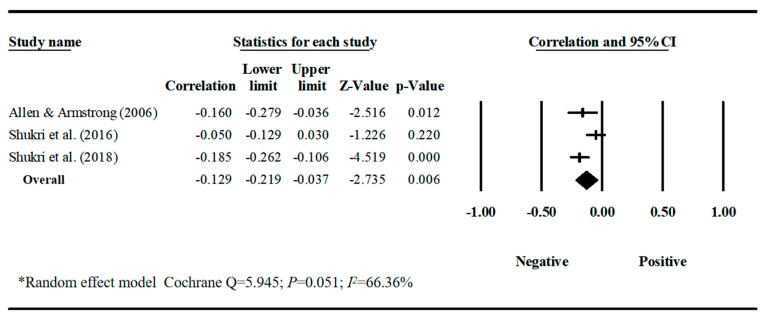
Forest plot of the correlation coefficients between work–family conflict and healthy diet. Note: Black squares/crosses indicate study-specific correlation coefficients, horizontal lines represent 95% confidence intervals, and the black diamond denotes the overall pooled effect size estimated using a random-effects model. Data sourced from the following studies: (Allen & Armstrong, 2006; Shukri et al., 2016, 2018).

**Figure 4:**
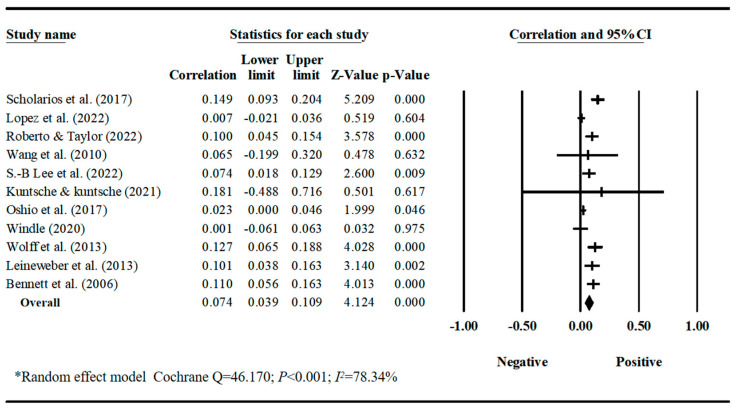
Forest plot of the correlation coefficients between work–family conflict and alcohol consumption. Note: Black squares/crosses indicate study-specific correlation coefficients, horizontal lines represent 95% confidence intervals, and the black diamond denotes the overall pooled effect size estimated using a random-effects model. Data sourced from the following studies: (Scholarios et al., 2017; Lopez et al., 2022; Roberto & Taylor, 2022; Wang et al., 2010; S.-B. Lee et al., 2022; Kuntsche & Kuntsche, 2021; Oshio et al., 2017; Windle, 2020; Wolff et al., 2013; Leineweber et al., 2013; Bennett et al., 2006).

**Figure 5:**
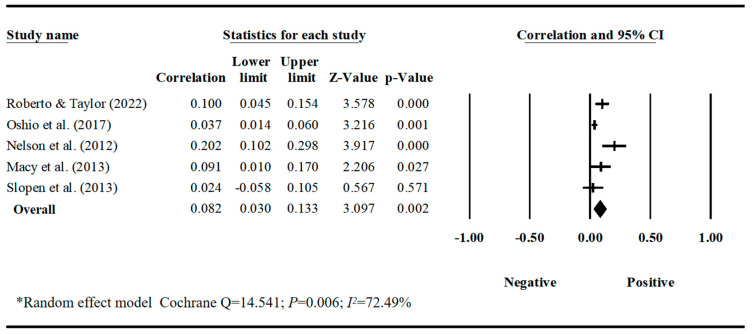
Forest plot of the correlation coefficients between work–family conflict and smoking behaviors. Note: Black squares/crosses indicate study-specific correlation coefficients, horizontal lines represent 95% confidence intervals, and the black diamond denotes the overall pooled effect size estimated using a random-effects model. Data sourced from the following studies: (Roberto & Taylor, 2022; Oshio et al., 2017; Nelson et al., 2012; Macy et al., 2013; Slopen et al., 2013).

**Figure 6:**
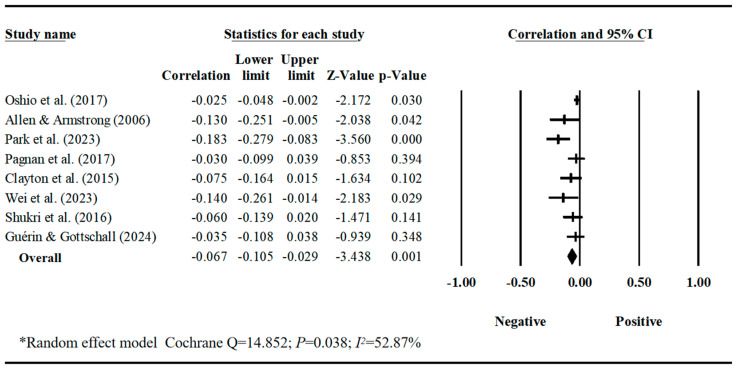
Forest plot of the correlation coefficients between work–family conflict and physical activity levels. Note: Black squares/crosses indicate study-specific correlation coefficients, horizontal lines represent 95% confidence intervals, and the black diamond denotes the overall pooled effect size estimated using a random-effects model. Data sourced from the following studies: (Oshio et al., 2017; Allen & Armstrong, 2006; Park et al., 2023; Pagnan et al., 2017; Clayton et al., 2015; Wei et al., 2023; Shukri et al., 2016; Guérin & Gottschall, 2024).

Our meta-analyses revealed behavior-specific associations. Work–family conflict had a small but significant positive correlation with sleep disturbances, with a pooled correlation coefficient of 0.188 (95% CI = 0.128, 0.247, *k* = 12) (Table 1, Figure 2). Work–family conflict was negatively correlated with healthy diet, with a pooled correlation coefficient of −0.129 (95% CI = −0.219, −0.037, *k* = 3). The estimate for healthy diet should be interpreted with caution due to the small number of studies and limited sample diversity. (Table 1, Figure 3). Work–family conflict had a very small, yet statistically significant, correlation with alcohol consumption ($\bar{r}$ = 0.074, 95% CI = 0.039, 0.109, *k* = 11) (Table 1, Figure 4), smoking behaviors ($\bar{r}$ = 0.082, 95% CI = 0.03, 0.133, *k* = 5) (Table 1, Figure 5), and physical activity levels ($\bar{r}$ = −0.075, 95% CI = −0.11, −0.03, *k* = 8) (Table 1, Figure 6).

The assessment of publication bias revealed slight funnel plot asymmetry for alcohol consumption and physical activity levels (Figures S1–S5), confirmed by a weak but significant Egger regression test. Trim-and-fill analyses reduced the overall effect size of the relationship between work–family conflict and alcohol consumption (*r* = 0.065; 95% CI, 0.031, 0.098) while maintaining its significance. However, the point estimate of the relationship between work–family conflict and physical activity levels was attenuated (*r* = −0.032; 95% CI, −0.072, 0.008) and became non-significant after adjustment. It should be noted that the pooled estimate for healthy diet was based on only three studies. Although the association reached statistical significance, this finding should be interpreted with caution due to the small number of available effect sizes and the limited generalizability of the samples.

## 4. Discussion

This study provides the first systematic synthesis and meta-analysis of the relationship between work–family conflict and multiple health behaviors. By quantifying the magnitude of these associations across five behavioral domains, our findings bridge the management and occupational health psychology literature with public health research—demonstrating that work–family conflict is not merely a workplace productivity issue, but a fundamental determinant of individuals’ daily health practices.

Engaging in health behaviors is associated with improved mental health and the prevention of the onset and progression of many chronic diseases (e.g., cardiovascular disease, diabetes, cancer) (Basten et al., 2024; Hutchesson et al., 2025; Liu et al., 2012; Silva et al., 2024). Work and family are the primary contexts in which contemporary individuals are engaged on a daily basis, and the conflict between work and family domains can influence individuals’ ability and motivation to adopt and sustain health behaviors in their daily lives (Gooding et al., 2020). Our meta-analysis revealed that work–family conflict was associated with sleep disturbances and healthy diet, and mildly associated with alcohol consumption, smoking behaviors, and reduced physical activity levels.

The present study found a small-to-medium-sized positive association between work–family conflict and sleep disturbances. This association may be explained by two theories. From a “stress theory” perspective, when individuals find it hard to reconcile multiple roles across work and family domains, they may experience chronic stress, which in turn can disrupt the initial stages of sleep (e.g., difficulty falling asleep) or lead to nocturnal awakenings due to elevated physiological and psychological activation (Magee et al., 2018). This chronic stress may also manifest as somatization symptoms (e.g., skeletal muscle pain) (Kim et al., 2013), which can further disrupt sleep quality. Additionally, from the other perspective of Conservation of Resources (COR) theory, time is a finite resource. To meet the dual demands of work and family, individuals may need to sacrifice sleep time (e.g., staying up late to work overtime or handle household chores), which directly leads to insufficient sleep duration and disrupts the sleep–wake rhythm (Barnes et al., 2012).

This study also identified a negative association between work–family conflict and healthy diet. Research has indicated that people may turn to food as a coping mechanism for emotional stress (Macht & Simons, 2000). Individuals who perceived greater stress may be more likely to consume fatty foods while neglecting fruits and vegetables (Cartwright et al., 2003). Work–family conflict can exacerbate stress levels, triggering stress-induced responses which can drive individuals to choose unhealthy eating patterns as a form of comfort (Shukri et al., 2018). Additionally, preparing healthy meals is often perceived as time-consuming and labor-intensive. Individuals experiencing work–family conflict may find it challenging to make an effort to prepare meals with sufficient fruits, vegetables, and fiber, given that the conflict has already consumed a significant portion of their time and energy (Allen & Armstrong, 2006).

The present study revealed a modest yet positive association of work–family conflict with smoking behaviors and alcohol consumption. Work–family conflict can induce anxiety, depression, and frustration, and nicotine and alcohol consumption may be employed as quick stress relievers to suppress negative emotions (e.g., through dopamine release), thereby generating a short-term illusion of “relaxation” (Roberto & Taylor, 2022). When individuals feel overwhelmed, the calming effect of alcohol or the short-lived sense of focus from smoking may serve as a means of escape (Kuntsche & Kuntsche, 2021). Notably, the correlation between work–family conflict and alcohol consumption could be affected by publication bias. Nevertheless, sensitivity analyses using trim-and-fill methods indicated that, although the effect size of this correlation was attenuated, it still remained statistical significance.

Although the majority of included samples had balanced gender distributions, gender may still moderate the relationship between work–family conflict and health behaviors. The predominance of gender-balanced samples in the literature is encouraging, as it enhances the generalizability of findings across sexes. However, statistical balance in sample composition does not necessarily imply that the effects of work–family conflict are experienced or expressed similarly by men and women. Women continue to bear disproportionate childcare and domestic responsibilities in many societies, which may amplify the time-based depletion effects of work–family conflict on sleep and diet. A recent Swedish cohort study found that the mental health consequences of work–family conflict were more pronounced among mothers than fathers, particularly in households with young children (Taouk et al., 2025). Extending this logic to behavioral outcomes, women experiencing high work–family conflict may have less time and energy for exercise and healthy meal preparation than their male counterparts, potentially widening gender disparities in health behaviors. Future research should explicitly test whether the associations observed in this meta-analysis differ by gender and parental status, and whether these differences are mediated by unequal domestic labor distribution.

The relationship between work–family conflict and physical activity levels appears to be inconsistent. Although the meta-analysis showed a small negative correlation between work–family conflict and physical activity levels, the Egger regression tests indicated that this correlation is subject to publication bias. After adjusting for this bias, the correlation became non-significant. Despite the fact that the dual demands of work and family may reduce time and decrease motivation for exercise (Allen & Armstrong, 2006), this impact of work–family conflict on physical activity levels seems to be mild or negligible.

It is worth noting that the magnitude of the association between work–family conflict and health behaviors is generally small and differs by types of health behaviors. It is the presence of work–family conflict which may deplete time and energy resources, thereby increasing risk of sleep disturbances and unhealthy diets (Scholarios et al., 2017). However, smoking behaviors, alcohol consumption and physical activity levels appear to be only mildly related to work–family conflict, as these behaviors are influenced by multiple factors at multiple levels, including habits, stress, motivations, and social and environmental factors (Gardner, 2015; Sallis et al., 2016). In particular, the finding regarding healthy diet warrants replication in more diverse occupational and cultural contexts, given the small number of studies contributing to this estimate.

From a practical standpoint, our findings support several concrete organizational strategies. First, flexible work arrangements (e.g., flextime, compressed workweeks) can help employees regain temporal autonomy, thereby reducing time-based work–family conflict and preserving opportunities for sleep and meal preparation. Second, supervisor support training programs have been shown to enhance family-supportive supervisor behaviors (FSSBs), which buffer the negative effects of work–family conflict on sleep and emotional exhaustion. Third, workplace wellness initiatives that explicitly address work–family stress—such as on-site healthy food options or designated physical activity breaks—may translate the modest statistical associations observed in this meta-analysis into meaningful behavioral change. These recommendations align with COR theory’s principle of resource replacement: When work–family conflict depletes resources, organizations can help employees replenish them through structural and social supports.

## 5. Limitations

Several limitations of the study should be considered when interpreting the results. Firstly, up to 70% of the effect sizes in the meta-analysis used a cross-sectional design, which limits causal inference and precludes conclusions about long-term effects. Secondly, the work–family conflict and health behavior measures used in the included studies varied, and instruments to objectively measure health behaviors were lacking, which in turn may have biased the analysis. Third, publication bias was identified in several correlations. To address this, we suggest searching the gray literature to include the relevant unpublished studies, thereby providing a more comprehensive evaluation of the effect sizes. Third, due to the limited number of studies reporting effect sizes separately for work→family and family→work conflict, we collapsed these two directions into a single composite measure. Although this approach maximizes statistical power, it may obscure potentially distinct relationships between conflict direction and specific health behaviors. Future research should examine whether the direction of conflict moderates behavioral outcomes.

## 6. Conclusions

This study is original in conducting a systematic review of studies regarding the association between work–family conflict and health behaviors. In addition, meta-analyses were used to derive pooled effect sizes, which provided a comprehensive understanding of the correlations between work–family conflict and different types of health behaviors. The findings revealed that work–family conflict is moderately associated with sleep disturbances and mildly associated with healthy diets. However, the association of work–family conflict with smoking behaviors, alcohol consumption, and physical activity levels appears to be small or non-significant. The findings of this study suggest that reducing work–family conflict—whether through organizational policy changes, family-level support, or individual coping strategies—may be a promising avenue for promoting healthy sleep and dietary behaviors. However, the cross-sectional nature of most included studies precludes causal inference. Future research using longitudinal and intervention designs is needed to determine which specific strategies are most effective and for whom.

## Figures and Tables

**Table 1:** Correlation between work–family conflict and health behaviors.

Types of Health Behaviors	Studies, *k*	Pooled Correlation Coefficient, Value (95% CI)
Sleep disturbances	12	0.188 (0.128, 0.247)
Alcohol consumption	11	0.074 (0.039, 0.109)
Smoking behaviors	5	0.082 (0.03, 0.133)
Physical activity levels	8	−0.067 (−0.105, −0.029)
Healthy diet	3	−0.129 (−0.219, −0.037)

## Data Availability

Data are contained within the article. The data presented in this study can be requested from the authors.

## Supplementary Materials

The following supporting information can be downloaded at: https://www.mdpi.com/article/10.3390/bs16030386/s1, Figures S1–S5: Funnel plot of the correlation between work–family conflict and health behaviors; Table S1: PRISMA (Preferred Reporting Items for Systematic Reviews and Meta-Analyses) 2020 checklist; Table S2: Search strategy; Table S3: Characteristics of the included studies; Table S4: Risk of bias assessment for the included studies; Table S5: The relationship between Work–family conflict and health behaviors in included studies. The following references are cited in the Supplementary Material (Table S3): Carlson & Frone, 2003; Chandola et al., 2004; Elo et al., 2000; Frone, 2000; Groesz et al., 2012; Grzywacz, 2000; Grzywacz & Marks, 2000; Gutek et al., 1991; Harvey et al., 2008; Kelloway et al., 1999; Kopelman et al., 1983; Marshall & Barnett, 1993; Mohr et al., 2005; Mojza et al., 2010; Pasman & Thompson, 1988; Patterson et al., 2005; Pitsopoulos & Greenwood, 2002; Schat et al., 2005; Wilsnack et al., 1991; Yu et al., 2011.
