# Supplementary material for: The Relationship Between Work–Family Conflict and Health Behaviors: A Systematic Review and Meta-Analysis
Source: Behav Sci (Basel). 2026 Mar 7;16(3):386. doi: 10.3390/bs16030386 (PMC13024141; doi:10.3390/bs16030386)
Supplement: Supplementary file 1 [file behavsci-16-00386-s001.zip › behavsci-4053103-supplementary.pdf]

Table S1. PRISMA (Preferred Reporting Items for Systematic Reviews and Meta-Analyses) 2020 checklist.

PRISMA 2020 Main Checklist

Topic	No.	Item	Location where item is reported
**TITLE**			
**Title**	1	Identify the report as a systematic review.	p.1
**ABSTRACT**			
**Abstract**	2	See the PRISMA 2020 for Abstracts checklist	
**INTRODUCTION**			
**Rationale**	3	Describe the rationale for the review in the context of existing knowledge.	p.3-4
**Objectives**	4	Provide an explicit statement of the objective(s) or question(s) the review addresses.	p.4
**METHODS**			
**Eligibility criteria**	5	Specify the inclusion and exclusion criteria for the review and how studies were grouped for the syntheses.	p.5-6
**Information sources**	6	Specify all databases, registers, websites, organisations, reference lists and other sources searched or consulted to identify studies. Specify the date when each source was last searched or consulted.	p.5
**Search strategy**	7	Present the full search strategies for all databases, registers and websites, including any filters and limits used.	Appendix 2
**Selection process**	8	Specify the methods used to decide whether a study met the inclusion criteria of the review, including how many reviewers screened each record and each report retrieved, whether they worked independently, and if applicable, details of automation tools used in the process.	p.6
**Data collection process**	9	Specify the methods used to collect data from reports, including how many reviewers collected data from each report, whether they worked independently, any processes for obtaining or confirming data from study investigators, and if applicable, details of automation tools used in the process.	p.6

Topic	No.	Item	Location where item is reported
**Data items**	10a	List and define all outcomes for which data were sought. Specify whether all results that were compatible with each outcome domain in each study were sought (e.g. for all measures, time points, analyses), and if not, the methods used to decide which results to collect.	p.6
	10b	List and define all other variables for which data were sought (e.g. participant and intervention characteristics, funding sources). Describe any assumptions made about any missing or unclear information.	p.6
**Study risk of bias assessment**	11	Specify the methods used to assess risk of bias in the included studies, including details of the tool(s) used, how many reviewers assessed each study and whether they worked independently, and if applicable, details of automation tools used in the process.	p.6-7
**Effect measures**	12	Specify for each outcome the effect measure(s) (e.g. risk ratio, mean difference) used in the synthesis or presentation of results.	p.7
**Synthesis methods**	13a	Describe the processes used to decide which studies were eligible for each synthesis (e.g. tabulating the study intervention characteristics and comparing against the planned groups for each synthesis (item 5)).	p.7-8
	13b	Describe any methods required to prepare the data for presentation or synthesis, such as handling of missing summary statistics, or data conversions.	p.7-8
	13c	Describe any methods used to tabulate or visually display results of individual studies and syntheses.	p.7-8
	13d	Describe any methods used to synthesize results and provide a rationale for the choice(s). If meta-analysis was performed, describe the model(s), method(s) to identify the presence and extent of statistical heterogeneity, and software package(s) used.	p.7-8
	13e	Describe any methods used to explore possible causes of heterogeneity among study results (e.g. subgroup analysis, meta-regression).	p.7-8
	13f	Describe any sensitivity analyses conducted to assess robustness of the synthesized results.	p.7-8
**Reporting bias assessment**	14	Describe any methods used to assess risk of bias due to missing results in a synthesis (arising from reporting biases).	p.7
**Certainty assessment**	15	Describe any methods used to assess certainty (or confidence) in the body of evidence for an outcome.	p.7-8

Topic	No.	Item	Location where item is reported
**RESULTS**			
**Study selection**	16a	Describe the results of the search and selection process, from the number of records identified in the search to the number of studies included in the review, ideally using a flow diagram.	Figure 1
	16b	Cite studies that might appear to meet the inclusion criteria, but which were excluded, and explain why they were excluded.	Figure 1
**Study characteristics**	17	Cite each included study and present its characteristics.	Appendix 3
**Risk of bias in studies**	18	Present assessments of risk of bias for each included study.	Appendix 4
**Results of individual studies**	19	For all outcomes, present, for each study: (a) summary statistics for each group (where appropriate) and (b) an effect estimate and its precision (e.g. confidence/credible interval), ideally using structured tables or plots.	Appendix 3
**Results of syntheses**	20a	For each synthesis, briefly summarise the characteristics and risk of bias among contributing studies.	p.9
	20b	Present results of all statistical syntheses conducted. If meta-analysis was done, present for each the summary estimate and its precision (e.g. confidence/credible interval) and measures of statistical heterogeneity. If comparing groups, describe the direction of the effect.	Table I
	20c	Present results of all investigations of possible causes of heterogeneity among study results.	Figure 2-6
	20d	Present results of all sensitivity analyses conducted to assess the robustness of the synthesized results.	p.11
**Reporting biases**	21	Present assessments of risk of bias due to missing results (arising from reporting biases) for each synthesis assessed.	Appendix 6
**Certainty of evidence**	22	Present assessments of certainty (or confidence) in the body of evidence for each outcome assessed.	p.11
**DISCUSSION**			
**Discussion**	23a	Provide a general interpretation of the results in the context of other evidence.	p.11
	23b	Discuss any limitations of the evidence included in the review.	p.14
	23c	Discuss any limitations of the review processes used.	p.14
	23d	Discuss implications of the results for practice, policy, and future research.	p.14-15

Topic	No.	Item	Location where item is reported
**OTHER INFORMATION**			
**Registration and protocol**	24a	Provide registration information for the review, including register name and registration number, or state that the review was not registered.	N/A
	24b	Indicate where the review protocol can be accessed, or state that a protocol was not prepared.	N/A
	24c	Describe and explain any amendments to information provided at registration or in the protocol.	N/A
**Support**	25	Describe sources of financial or non-financial support for the review, and the role of the funders or sponsors in the review.	Title page
**Competing interests**	26	Declare any competing interests of review authors.	Title page
**Availability of data, code and other materials**	27	Report which of the following are publicly available and where they can be found: template data collection forms; data extracted from included studies; data used for all analyses; analytic code; any other materials used in the review.	N/A

PRISMA Abstract Checklist

Topic	No.	Item	Reported?
**TITLE**			
**Title**	1	Identify the report as a systematic review.	Yes
**BACKGROUND**			
**Objectives**	2	Provide an explicit statement of the main objective(s) or question(s) the review addresses.	Yes
**METHODS**			
**Eligibility criteria**	3	Specify the inclusion and exclusion criteria for the review.	Yes
**Information sources**	4	Specify the information sources (e.g. databases, registers) used to identify studies and the date when each was last searched.	Yes
**Risk of bias**	5	Specify the methods used to assess risk of bias in the included studies.	Yes
**Synthesis of results**	6	Specify the methods used to present and synthesize results.	Yes
**RESULTS**			
**Included studies**	7	Give the total number of included studies and participants and summarise relevant characteristics of studies.	Yes
**Synthesis of results**	8	Present results for main outcomes, preferably indicating the number of included studies and participants for each. If meta-analysis was done, report the summary estimate and confidence/credible interval. If comparing groups, indicate the direction of the effect (i.e. which group is favoured).	Yes
**DISCUSSION**			
**Limitations of evidence**	9	Provide a brief summary of the limitations of the evidence included in the review (e.g. study risk of bias, inconsistency and imprecision).	Yes
**Interpretation**	10	Provide a general interpretation of the results and important implications.	Yes
**OTHER**			
**Funding**	11	Specify the primary source of funding for the review.	Yes
**Registration**	12	Provide the register name and registration number.	No

From: Page, M. J., McKenzie, J. E., Bossuyt, P. M., Boutron, I., Hoffmann, T. C., Mulrow, C. D., Shamseer, L., Tetzlaff, J. M., Akl, E. A., Brennan, S. E., Chou, R., Glanville, J., Grimshaw, J. M., Hróbjartsson, A., Lalu, M. M., Li, T., Loder, E. W., Mayo-Wilson, E., McDonald, S., … Moher, D. (2021). The PRISMA 2020 statement: An updated guideline for reporting systematic reviews. *BMJ (Clinical Research Ed.)*, *372*, n71. https://doi.org/10.1136/bmj.n71.

Table S2. Search strategy.

Database	Embase, Web of Science, PubMed, PsycINFO	
Search terms	MeSH term	Entry Terms
	work-life balance	Work-Life Conflict<br>Conflicts, Work-Life<br>Conflict, Work-Life<br>Work Life Conflict<br>Work-Life Conflicts<br>Life-Work Imbalance<br>Work Life Imbalance<br>Imbalances, Work Life<br>Imbalance, Work Life<br>Life Imbalances, Work<br>Life Imbalance, Work<br>Work Life Imbalances<br>Work-Family Balance<br>Balances, Work-Family<br>Balance, Work-Family<br>Work Family Balance<br>Work-Family Balances<br>Work-Family Conflict<br>Conflicts, Work-Family<br>Conflict, Work-Family<br>Work Family Conflict<br>Work-Family Conflicts
	Health behavior	Health Behaviors<br>Health-Related Behavior<br>Health Related Behavior<br>Health-Related Behaviors
	Heathy lifestyle	Healthy Life Styles<br>Healthy Lifestyles<br>Healthy Life Style
	Exercise	Exercises<br>Physical Activity<br>Physical Activities<br>Physical Exercise<br>Physical Exercises<br>Acute Exercise<br>Acute Exercises<br>Isometric Exercises<br>Isometric Exercise<br>Aerobic Exercise<br>Aerobic Exercises<br>Exercise Training<br>Exercise Trainings
	Sleep	Sleeping Habits

		Sleep Habits<br>Sleep Habit<br>Sleeping Habit
	Smoking	Smoking Behaviors<br>Smoking Behavior<br>Smoking Habit<br>Smoking Habits
	Eating	Food Intake<br>Macronutrient Intake<br>Macronutrient Intakes<br>Dietary Intake<br>Dietary Intakes<br>Micronutrient Intake<br>Micronutrient Intakes<br>Ingestion<br>Feed Intake<br>Feed Intakes<br>Nutrient Intake<br>Nutrient Intakes<br>Nutritional Intake<br>Nutritional Intakes
	Healthy Diet	Healthy Diets<br>Healthy Eating<br>Healthy Nutrition<br>Prudent Diet<br>Prudent Diets<br>Healthy Eating Index<br>Healthy Eating Indices

Pubmed

#1 Search: (((((((((((((((work-life balance[MeSH Terms]) OR (Work-Life Conflict[Title/Abstract])) OR (Work Life Conflict[Title/Abstract])) OR (Work-Life Conflicts[Title/Abstract])) OR (Work-Family Balance[Title/Abstract])) OR (Work Family Balance[Title/Abstract])) OR (Work-Family Balances[Title/Abstract])) OR (Life-Work Imbalance[Title/Abstract]) "work life balance"[MeSH Terms] OR "work life conflict"[Title/Abstract] OR "work life conflict"[Title/Abstract] OR "work life conflicts"[Title/Abstract] OR "work family balance"[Title/Abstract] OR "work family balance"[Title/Abstract] OR "work family balances"[Title/Abstract] OR "life work imbalance"[Title/Abstract])) OR (Work-life enrichment[Title/Abstract])) OR (Work life enrichment[Title/Abstract])) OR (Work-family enrichment[Title/Abstract])) OR (Work family enrichment[Title/Abstract])) OR (Family-work conflict[Title/Abstract])) OR (Family work conflict[Title/Abstract])) OR (Family-work balance[Title/Abstract])) OR (Family work balance[Title/Abstract])

#2 Search: ((((Health behavior[MeSH Terms]) OR (Health Behaviors[Title/Abstract])) OR (Health-Related Behavior[Title/Abstract])) OR (Health Related Behavior[Title/Abstract])) OR (Health-Related Behaviors[Title/Abstract])

#3 Search: ((((Heathy lifestyle[MeSH Terms])) OR (Healthy Life Styles[Title/Abstract])) OR (Healthy Lifestyles[Title/Abstract])) OR (Healthy Life Style[Title/Abstract])

#4 Search: (((((((((((((Exercise[MeSH Terms])) OR (Exercises[Title/Abstract])) OR (Physical Activity[Title/Abstract])) OR (Physical Activities[Title/Abstract])) OR (Physical Exercise[Title/Abstract])) OR (Physical Exercises[Title/Abstract])) OR (Acute Exercise[Title/Abstract])) OR (Acute Exercises[Title/Abstract])) OR (Isometric Exercises[Title/Abstract])) OR (Isometric Exercise[Title/Abstract])) OR (Aerobic Exercise[Title/Abstract])) OR (Aerobic Exercises[Title/Abstract])) OR (Exercise Training[Title/Abstract])) OR (Exercise Trainings[Title/Abstract])

#5 Search: ((((((Self-examination[MeSH Terms])) OR (Self-Examinations[Title/Abstract])) OR (Examination, Self[Title/Abstract])) OR (Examinations, Self[Title/Abstract])) OR (Self Examination[Title/Abstract])) OR (Self Examinations[Title/Abstract])

#6 Search: (((((((Sleep[MeSH Terms])) OR (Sleeping Habits[Title/Abstract])) OR (Sleep Habits[Title/Abstract])) OR (Sleep Habit[Title/Abstract])) OR (Sleeping Habit[Title/Abstract])) OR (Sleep Quality[Title/Abstract])) OR (Sleep Hygiene[Title/Abstract])

#7 Search: ((((Smoking[MeSH Terms]) OR (Smoking Behaviors[Title/Abstract])) OR (Smoking Behavior[Title/Abstract])) OR (Smoking Habit[Title/Abstract])) OR (Smoking Habits[Title/Abstract])

#8 Search: ((((((((((((((Eating[MeSH Terms])) OR (Food Intake[Title/Abstract])) OR (Macronutrient Intake[Title/Abstract])) OR (Macronutrient Intakes[Title/Abstract])) OR (Dietary Intake[Title/Abstract])) OR (Dietary Intakes[Title/Abstract])) OR (Micronutrient Intake[Title/Abstract])) OR (Micronutrient Intakes[Title/Abstract])) OR (Ingestion[Title/Abstract])) OR (Feed Intake[Title/Abstract])) OR (Feed Intakes[Title/Abstract])) OR (Nutrient Intake[Title/Abstract])) OR (Nutrient Intakes[Title/Abstract])) OR (Nutritional Intake[Title/Abstract])) OR (Nutritional Intakes[Title/Abstract])

#9 Search: ((((((((Healthy Diet[MeSH Terms]) OR (Healthy Diets[Title/Abstract])) OR (Healthy Eating[Title/Abstract])) OR (Healthy Nutrition[Title/Abstract])) OR (Prudent Diet[Title/Abstract])) OR (Prudent Diets[Title/Abstract])) OR (Healthy Eating Index[Title/Abstract])) OR (Healthy Eating Indices[Title/Abstract])

#10 Search: "exergaming"[MeSH Terms] OR "gymnastics"[MeSH Terms] OR "walking"[MeSH Terms]OR " running "[MeSH Terms] OR "swimming"[MeSH Terms]

Web of Science

#1 (((((((((((((((TS=("work-life balance")) OR TS=("Work-Life Conflict"))) OR TS=("Work Life Conflict")) OR TS=("Work-Life Conflicts")) OR TS=("Work-Family Balance")) OR TS=("Work Family Balance")) OR TS=("Work-Family Balances")) OR TS=("Life-Work Imbalance")) OR TS=("Work-life enrichment")) OR TS=("Work life enrichment")) OR TS=("Work-family enrichment")) OR TS=("Work family enrichment")) OR TS=("Family-work conflict")) OR TS=("Family work conflict")) OR TS=("Family-work balance")) OR TS=("Family work balance")

#2 ((((TS=("Health behavior")) OR TS=("Health Behaviors")) OR TS=("Health-Related Behavior")) OR TS=("Health Related Behavior")) OR TS=("Health-Related Behaviors")

#3 (((TS=(“Heathy lifestyle”)) OR TS=(“Healthy Life Styles”)) OR TS=(“Healthy Lifestyles”)) OR TS=(“Healthy Life Style”)

#4 (((((TS=(“Self-examination”)) OR TS=(“Self-Examinations”)) OR TS=(“Examination, Self”)) OR TS=(“Examinations, Self”)) OR TS=(“Self Examination”)) OR TS=(“Self Examinations”)

#5 (((((((((((((TS=(“Exercise”)) OR TS=(“Exercises”)) OR TS=(“Physical Activity”))) OR TS=(“Physical Activities”)) OR TS=(“Physical Exercise”)) OR TS=(“Physical Exercises”)) OR TS=(“Acute Exercise”)) OR TS=(“Acute Exercises”)) OR TS=(“Isometric Exercises”)) OR TS=(“Isometric Exercise”)) OR TS=(“Aerobic Exercise”)) OR TS=(“Exercise Training”)) OR TS=(“Exercise Trainings”)) OR TS=(“Aerobic Exercises”)

#6 ((((((TS=(“Sleep”)) OR TS=(“Sleeping Habits”)) OR TS=(“Sleep Habits”)) OR TS=(“Sleep Habit”)) OR TS=(“Sleeping Habit”)) OR TS=(“Sleep Hygiene”)) OR TS=(“Sleep Quality”)

#7 ((((TS=(“Smoking”)) OR TS=(“Smoking Behaviors”)) OR TS=(“Smoking Behavior”)) OR TS=(“Smoking Habit”)) OR TS=(“Smoking Habits”)

#8 ((((((((((((((TS=(“Eating”)) OR TS=(“Food Intake”)) OR TS=(“Macronutrient Intake”)) OR TS=(“Macronutrient Intakes”)) OR TS=(“Dietary Intake”)) OR TS=(“Dietary Intakes”)) OR TS=(“Micronutrient Intake”)) OR TS=(“Micronutrient Intakes”)) OR TS=(“Ingestion”)) OR TS=(“Feed Intake”)) OR TS=(“Feed Intakes”)) OR TS=(“Nutrient Intake”)) OR TS=(“Nutrient Intakes”)) OR TS=(“Nutritional Intake”)) OR TS=(“Nutritional Intakes”)) OR TS=(“Drinking”)

#9 (((((((TS=(“Healthy Diet”)) OR TS=(“Healthy Diets”)) OR TS=(“Healthy Eating”)) OR TS=(“Healthy Nutrition”)) OR TS=(“Prudent Diet”)) OR TS=(“Prudent Diets”)) OR TS=(“Healthy Eating Index”)) OR TS=(“Healthy Eating Indices”)

#10 ((((TS=(“exergaming”)) OR TS=(“gymnastics”)) OR TS=(“walking”)) OR TS=(“running”)) OR TS=(“swimming”)

Embase

#1 'work-life balance'/exp OR 'work-life conflict':ti,ab,kw OR 'work life conflict':ti,ab,kw OR 'work-life conflicts':ti,ab,kw OR 'work-family balance':ti,ab,kw OR 'work family balance':ti,ab,kw OR 'work-family balances':ti,ab,kw OR 'life-work imbalance':ti,ab,kw OR 'work-life enrichment':ti,ab,kw OR 'work life enrichment':ti,ab,kw OR 'work-family enrichment':ti,ab,kw OR 'work family enrichment':ti,ab,kw OR 'family-work conflict':ti,ab,kw OR 'family work conflict':ti,ab,kw OR 'family-work balance':ti,ab,kw OR 'family work balance':ti,ab,kw

#2 'health behavior'/exp OR 'health behaviors':ti,ab,kw OR 'health-related behavior':ti,ab,kw OR 'health related behavior':ti,ab,kw OR 'health-related behaviors':ti,ab,kw

#3 'heathy lifestyle' OR 'healthy life styles':ti,ab,kw OR 'healthy lifestyles':ti,ab,kw OR 'healthy life style':ti,ab,kw

#4 'self examination'/exp OR 'examination, self':ti,ab,kw OR 'examinations, self':ti,ab,kw OR 'self examination':ti,ab,kw OR 'self examinations':ti,ab,kw

#5 'exercise'/exp OR exercises:ti,ab,kw OR 'physical activity':ti,ab,kw OR 'physical activities':ti,ab,kw OR 'physical exercise':ti,ab,kw OR 'physical exercises':ti,ab,kw OR 'acute exercise':ti,ab,kw OR 'acute exercises':ti,ab,kw OR 'isometric exercises':ti,ab,kw OR 'isometric exercise':ti,ab,kw OR 'aerobic exercise':ti,ab,kw OR 'aerobic exercises':ti,ab,kw OR 'exercise training':ti,ab,kw OR 'exercise trainings':ti,ab,kw

#6 'sleep'/exp OR 'sleeping habits':ti,ab,kw OR 'sleep habits':ti,ab,kw OR 'sleep habit':ti,ab,kw OR 'sleeping habit':ti,ab,kw OR 'sleep hygiene':ti,ab,kw OR 'sleep quality':ti,ab,kw

#7 'smoking'/exp OR 'smoking behaviors':ti,ab,kw OR 'smoking behavior':ti,ab,kw OR 'smoking habit':ti,ab,kw OR 'smoking habits':ti,ab,kw

#8 'eating'/exp OR 'food intake':ti,ab,kw OR 'macronutrient intake':ti,ab,kw OR 'macronutrient intakes':ti,ab,kw OR 'dietary intake':ti,ab,kw OR 'dietary intakes':ti,ab,kw OR 'micronutrient intake':ti,ab,kw OR 'micronutrient intakes':ti,ab,kw OR ingestion:ti,ab,kw OR 'feed intake':ti,ab,kw OR 'feed intakes':ti,ab,kw OR 'nutrient intake':ti,ab,kw OR 'nutrient intakes':ti,ab,kw OR 'nutritional intake':ti,ab,kw OR 'nutritional intakes':ti,ab,kw OR drinking:ti,ab,kw

#9 'healthy diet'/exp OR 'healthy diets':ti,ab,kw OR 'healthy eating':ti,ab,kw OR 'healthy nutrition':ti,ab,kw OR 'prudent diet':ti,ab,kw OR 'prudent diets':ti,ab,kw OR 'healthy eating index':ti,ab,kw OR 'healthy eating indices':ti,ab,kw

#10 'exergaming'/exp OR 'gymnastics'/exp OR 'walking'/exp OR 'running'/exp OR 'swimming'/exp

PsycINFO

#1 AB work-life balance OR AB Work-Life Conflict OR AB Work Life Conflict OR AB Work-Life Conflicts OR AB Work-Family Balance OR AB Work Family Balance OR AB Work-Family Balances OR AB Life-Work Imbalance OR AB Work-life enrichment OR AB Work life enrichment OR AB Work-family enrichment OR AB Work family enrichment OR AB Family-work conflict OR AB Family work conflict OR AB Family-work balance OR AB Family work balance

#2 AB Health Behaviors OR AB Health behavior OR AB Health-Related Behavior OR AB Health Related Behavior OR AB Health-Related Behaviors

#3 AB Heathy lifestyle OR AB Healthy Life Styles OR AB Healthy Lifestyles OR AB Healthy Life Style

#4 AB Self-examination OR AB Self-Examinations OR AB Examination, Self OR AB Examinations, Self OR AB Self Examination OR AB Self Examinations

#5 AB Exercise OR AB Exercises OR AB Physical Activity OR AB Physical Activities OR AB Physical Exercise OR AB Physical Exercises OR AB Acute Exercise OR AB Acute Exercises OR AB Isometric Exercises OR AB Isometric Exercise OR AB Aerobic Exercise OR AB Aerobic Exercises OR AB Exercise Training OR AB Exercise Trainings

#6 AB Sleep OR AB Sleeping Habits OR AB Sleep Habits OR AB Sleep Habit OR AB Sleeping Habit OR AB Sleep Hygiene OR AB Sleep Quality

#7 AB Smoking OR AB Smoking Behaviors OR AB Smoking Behavior OR AB Smoking Habit OR AB Smoking Habits

#8 AB Eating OR AB Food Intake OR AB Macronutrient Intake OR AB Macronutrient Intakes OR AB Dietary Intake OR AB Dietary Intakes OR AB Micronutrient Intake OR AB Micronutrient Intakes OR AB Ingestion OR AB Feed Intake OR AB Feed Intakes OR AB Nutrient Intake OR AB Nutrient Intakes OR AB Nutritional Intake OR AB Nutritional Intakes OR AB Drinking

#9 AB Healthy Diet OR AB Healthy Diets OR AB Healthy Eating OR AB Healthy Nutrition OR AB Prudent Diet OR AB Prudent Diets OR AB Healthy Eating Index OR AB Healthy Eating Indices

#10 AB exergaming OR AB gymnastics OR AB walking OR AB running OR AB swimming

Table S3. Characteristics of the included studies.

Author (year)	country/region	Study design	Sample size	Mean age	Sex ratio	Occupation	Role in family	Measurement of work-family interplay	Types of health behaviors	Measurement of health behaviors	Risk of bias
(Zhang et al., 2017)	United States	cross-sectional design	397	43.15	94.5% females	Nurse	-	WFC-(Kopelman et al., 1983)	Sleep disturbances	PROMIS Sleep Disturbance -(Yu et al., 2011)	21
(Oshio et al., 2017)	Japan	longitudinal design	7551	-	21.2% females	Working in firm	-	WFC-(Chandola et al., 2004)	Current smoking, problem drinking, leisure-time physical inactivity	5 self-reported items	22
(Kuntsche & Kuntsche, 2021)	Switzerland	cross-sectional design	305	38.7	52.1% females	mixed	parents of pre-school children	WFC-(Netemeyer et al., 1996)	Alcohol use	A self-developed question	21
(Shukri et al., 2018)	Malaysia	cross-sectional design	586	36	60.8% females	mixed	parents	WFC-(Netemeyer et al., 1996)	Unhealthy eating	Unhealthy eating- (Groesz et al., 2012)	19
(Allen & Armstrong, 2006)	United States	cross-sectional design	246	38.6	78% females	mixed	Be married (or living with their partner) and/or have at least one child	WFC-(Carlson et al., 2000)	Physical activity, fatty food consumption, and healthy food consumption	fat foods - dietary fat screener healthy food - Behavioral Risk Factor Surveillance System State Questionnaire Physical activity- A self-	20

Author (year)	country/region	Study design	Sample size	Mean age	Sex ratio	Occupation	Role in family	Measurement of work-family interplay	Types of health behaviors	Measurement of health behaviors	Risk of bias
										developed question	
(Magee et al., 2018)	Australia	cross-sectional design	3170	40.8	45.5% females	mixed	parents	WFC/WFE-(Marshall & Barnett, 1993)	Sleep quality	7 self-reported items	22
(Makela et al., 2014)	Finland	longitudinal design	868	42.8	24% females	Mixed-with international business travel		WFC-(Carlson et al., 2000)	Sleep problem	Basic Nordic Sleep Questionnaire-(Partinen & Gislason, 1995)	20
(Eshak, 2019)	Egypt	cross-sectional design	1021	18-59	62.6% females	mixed		WFC-(Grzywacz & Marks, 2000)	Sleep disorders	Jenkins Sleep Questionnaire-(Jenkins et al., 1988)	22
(Aazami et al., 2016)	Malaysia	cross-sectional design	325	36.3	100% females	mixed	married	WFC-(Kelloway et al., 1999)	Sleep disturbances	Physical Health Questionnaire-(Schat et al., 2005)	21
(Shin & Jeong, 2020)	Korea	cross-sectional design	184	54.9	0% females	Truck drivers		WFC -6th European Working Conditions Survey (2015) Questionnaire	Sleep problem	6th European Working Conditions Survey (2015) Questionnaire	20
(Vleeshouwers et al., 2019)	Norwegia	longitudinal design	4681	44.6	55.1% females	mixed	single parents and married	WLC-General Nordic Questionnaire for Psychological and Social	Sleep problem	Sleep problem-(Harvey et al., 2008)	21

Author (year)	country/region	Study design	Sample size	Mean age	Sex ratio	Occupation	Role in family	Measurement of work-family interplay	Types of health behaviors	Measurement of health behaviors	Risk of bias
								Factors at Work -(Elo et al., 2000)			
(Lalluka et al., 2014)	Finland, Britain, Japan	longitudinal design	3881 3998 1834	-	48.9% females	mixed		WFC-(Grzywacz & Marks, 2000)	Sleep problem	Jenkins Sleep Questionnaire-(Jenkins et al., 1988), Pittsburgh Sleep Quality Index-(Buysse et al., 1989)	22
(Scholarios et al., 2017)	United Kingdom	cross-sectional design	1207	36-39	27% females	police officers		WFC-(Netemeyer et al., 1996)	Alcohol consumption, sleep disturbance	The standard shiftwork index-(Pitsopoulos & and Greenwood, 2002)	22
(Crain et al., 2014)	United States	cross-sectional design	623	46	39% females	information technology division		WFC-(Netemeyer et al., 1996)	Sleep quality and quantity	Pittsburgh Sleep Quality Index-(Buysse et al., 1989)	22
(Sekine et al., 2006)	Japan	cross-sectional design	3556	42.7	32.6% females	civil servants		WFC-(Chandola et al., 2004)	Sleep quality	Pittsburgh Sleep Quality Index-(Buysse et al., 1989)	22
(S. Lee et al., 2017)	United States	longitudinal design	102	45.2	46% females	information technology (IT) division		WFC-(Netemeyer et al., 1996)	Sleep quality, sleep hours, sleep latency	Pittsburgh Sleep Quality Index-(Buysse et al., 1989)	22
(Lopez et al., 2022)	United States	cross-sectional design	1228	37.2	50% females	STEM fields	married with	WFC-(Netemeyer et al., 1996)	Alcohol use	Alcohol Use Disorders Identification	21

Author (year)	country/region	Study design	Sample size	Mean age	Sex ratio	Occupation	Role in family	Measurement of work-family interplay	Types of health behaviors	Measurement of health behaviors	Risk of bias
							minor children			Test – Consumption-(Osaki et al., 2014)	
(Roberto & Taylor, 2022)	United States	cross-sectional design	1275	-	44% females	collar/front-line		WFC-(Frone, 2000)	Alcohol and cigarettes use	frequency of alcohol/cigarettes use-(Patterson et al., 2005)	18
(Wang et al., 2010)	China	longitudinal design	57	32.6	8% females	mixed		WFC-(Carlson & Frone, 2003)	Alcohol use	Alcohol consumption-(Mohr et al., 2005)	21
(S.-B. Lee et al., 2022)	Korea	longitudinal design	4615	46.7	46.7% females	mixed		Korea Institute for Health and Social Afairs (KIHASA)	Problem drinking	Alcohol Use Disorders Identification Test (AUDIT)	22
(Windle, 2020)	New York	longitudinal design	1004	17-33	53% females	mixed		WFC-(Frone, 2000)	Alcohol use	self-reported items	18
(Wolff et al., 2013)	Chicagoland	cross-sectional design	998	42	54.1% females	mixed	caregiver	WFC-(Kelloway et al., 1999)	Alcohol use	problematic alcohol use-(Wilsnack et al., 1991),	22
(Leineweber et al., 2013)	Swedish	longitudinal design	6580	48.6	56.1% females	mixed		A question that has been used in other studies	Problem drinking	CAGE-(Mayfield et al., 1974), AUDIT	22
(Bennett et al., 2006)	United States	cross-sectional design	1323	18-41+	32% females	mixed		WLC-(Frone, 2000)	At-risk drinking	the Institute of Behavioral Research (Texas Christian	19

Author (year)	country/region	Study design	Sample size	Mean age	Sex ratio	Occupation	Role in family	Measurement of work-family interplay	Types of health behaviors	Measurement of health behaviors	Risk of bias
										University）, CAGE	
(Nelson et al., 2012)	Boston	cross-sectional design	439	18-65+	82.5% females	mixed		Two questions from work and family researcher network	Current smoking	A self-developed question	21
(Macy et al., 2013)	Indiana	cross-sectional design	423	43.6	48.5% females	mixed	Married or have children	Midlife in the United States Study	Smoking quantity	A self-reported item	20
(Slopen et al., 2013)	United States	longitudinal design	4938	25-74	-	mixed		(Grzywacz, 2000)	Smoking behavior	A self-reported item	22
(Park et al., 2023)	Korea	cross-sectional design	373	38.6	60% females	mixed		WFC-(Kopelman et al., 1983)	Physical activity	physical activity level-(Mojza et al., 2010)	20
(Pagnan et al., 2017)	United States	cross-sectional design	811	41	44% females	mixed	parents	A self-developed question	Physical activity	A self-reported item	20
(Clayton et al., 2015)	United States	cross-sectional design	476	41.5	55% females	mixed		WFC-(Carlson et al., 2000)	Exercise	Exercise-(Pasman & Thompson, 1988)	20
(Wei et al., 2023)	China	cross-sectional design	243	34.8	48.3% females	mixed		WFC-(Carlson et al., 2000)	Exercise	Exercise-(Pasman & Thompson, 1988),	21
(Shukri et al., 2016)	UK, Malaysia	cross-sectional design	603	36.8	53.1% females	mixed		WFC-(Gutek et al., 1991)	Healthy diet, physical activity	A self-reported item	18

Author (year)	country/region	Study design	Sample size	Mean age	Sex ratio	Occupation	Role in family	Measurement of work-family interplay	Types of health behaviors	Measurement of health behaviors	Risk of bias
(Guérin & Gottschall, 2024)	Canadia	cross-sectional design	930	-	11.1% females	in hard sea occupations		WFC-(Netemeyer et al., 1996)	Physical activity	Leisure Time Exercise Questionnaire-(Godin, 2011)	20

Abbreviations: WFC Work-Family Conflict WLC Work-Life Conflict

Reference:

Aazami, S., Mozafari, M., Shamsuddin, K., & Akmal, S. (2016). Work-family conflict and sleep disturbance: The Malaysian working women study. *Industrial Health*, *54*(1), 50–57. https://doi.org/10.2486/indhealth.2015-0086

Allen, T. D., & Armstrong, J. (2006). Further examination of the link between work-family conflict and physical health—The role of health-related behaviors. *AMERICAN BEHAVIORAL SCIENTIST*, *49*(9), 1204–1221. https://doi.org/10.1177/0002764206286386

Bennett, J. B., Patterson, C. R., Wiitala, W. L., & Woo, A. (2006). Social risks for at-risk drinking in young workers: Application of work-life border theory. *JOURNAL OF DRUG ISSUES*, *36*(3), 485–513. https://doi.org/10.1177/002204260603600301

Buysse, D. J., Reynolds, C. F., Monk, T. H., Berman, S. R., & Kupfer, D. J. (1989). The Pittsburgh Sleep Quality Index: A new instrument for psychiatric practice and research. *Psychiatry Research*, *28*(2), 193–213. https://doi.org/10.1016/0165-1781(89)90047-4

Carlson, D. S., & Frone, M. R. (2003). Relation of Behavioral and Psychological Involvement to a New Four-Factor Conceptualization of Work-Family Interference. *Journal of Business and Psychology*, *17*(4), 515–535. https://doi.org/10.1023/A:1023404302295

Carlson, D. S., Kacmar, K. M., & Williams, L. J. (2000). Construction and Initial Validation of a Multidimensional Measure of Work–Family Conflict. *Journal of Vocational Behavior*, *56*(2), 249–276. https://doi.org/10.1006/jvbe.1999.1713

Chandola, T., Martikainen, P., Bartley, M., Lahelma, E., Marmot, M., Michikazu, S., Nasermoaddeli, A., & Kagamimori, S. (2004). Does conflict between home and work explain the effect of multiple roles on mental health? A comparative study of Finland, Japan, and the UK. *International Journal of Epidemiology*, *33*(4), 884–893. https://doi.org/10.1093/ije/dyh155

Clayton, R. W., Thomas, C. H., Singh, B., & Winkel, D. E. (2015). Exercise as a Means of Reducing Perceptions of Work-Family Conflict: A Test of the Roles of Self-Efficacy and Psychological Strain. *HUMAN RESOURCE MANAGEMENT*, *54*(6), 1013–1035. https://doi.org/10.1002/hrm.21611

Crain, T. L., Hammer, L. B., Bodner, T., Kossek, E. E., Moen, P., Lilienthal, R., & Buxton, O. M. (2014). Work-Family Conflict, Family-Supportive Supervisor Behaviors (FSSB), and Sleep Outcomes. *JOURNAL OF OCCUPATIONAL HEALTH PSYCHOLOGY*, *19*(2), 155–167. https://doi.org/10.1037/a0036010

Elo, A. L., Dallner, M., Gamberale, F., Hottinen, V., Knardahl, S., Lindström, K., Skogstad, A., & Orhede, E. (2000). Validation of the Nordic questionnaire for psychological and social factors at work -: QPSNordic. In M. Vartiainen, F. Avallone, & N. Anderson (Eds.), *INNOVATIVE THEORIES, TOOLS AND PRACTICES IN WORK AND ORGANIZATIONAL PSYCHOLOGY* (pp. 47–57). Hogrefe & Huber Publishers. https://webofscience.clarivate.cn/wos/alldb/full-record/WOS:000174050100004 (accessed on March 12, 2025)

Eshak, E. S. (2019). Work-to-family conflict rather than family-to-work conflict is more strongly associated with sleep disorders in Upper Egypt. *Industrial Health*, *57*(3), 351–358. https://doi.org/10.2486/indhealth.2018-0091

Frone, M. R. (2000). Interpersonal conflict at work and psychological outcomes: Testing a model among young workers. *Journal of Occupational Health Psychology*, *5*(2), 246–255. https://doi.org/10.1037//1076-8998.5.2.246

Godin, G. (2011). The Godin-Shephard Leisure-Time Physical Activity Questionnaire. *The Health & Fitness Journal of Canada*, *4*(1), 18-22. https://doi.org/10.14288/hfjc.v4i1.82

Groesz, L. M., McCoy, S., Carl, J., Saslow, L., Stewart, J., Adler, N., Laraia, B., & Epel, E. (2012). What is eating you? Stress and the drive to eat. *Appetite*, *58*(2), 717–721. https://doi.org/10.1016/j.appet.2011.11.028

Grzywacz, J. G. (2000). Work-family spillover and health during midlife: Is managing conflict everything? *American Journal of Health Promotion: AJHP*, *14*(4), 236–243. https://doi.org/10.4278/0890-1171-14.4.236

Grzywacz, J. G., & Marks, N. F. (2000). Reconceptualizing the work-family interface: An ecological perspective on the correlates of positive and negative spillover between work and family. *Journal of Occupational Health Psychology*, *5*(1), 111–126. https://doi.org/10.1037//1076-8998.5.1.111

Guérin, E., & Gottschall, S. (2024). Feeling the Strain: Understanding the Relationship between Stress and Physical Activity in Members of the Royal Canadian Navy. *Military Medicine*, *189*(9–10), 1857–1863. https://doi.org/10.1093/milmed/usae006

Gutek, B., Searle, S., & Klepa, L. (1991). Rational Versus Gender-Role Explanations for Work Family Conflict. *JOURNAL OF APPLIED PSYCHOLOGY*, *76*(4), 560–568. https://doi.org/10.1037/0021-9010.76.4.560

Harvey, A. G., Stinson, K., Whitaker, K. L., Moskovitz, D., & Virk, H. (2008). The subjective meaning of sleep quality: A comparison of individuals with and without insomnia. *Sleep*, *31*(3), 383–393. https://doi.org/10.1093/sleep/31.3.383

Jenkins, C. D., Stanton, B. A., Niemcryk, S. J., & Rose, R. M. (1988). A scale for the estimation of sleep problems in clinical research. *Journal of Clinical Epidemiology*, *41*(4), 313–321. https://doi.org/10.1016/0895-4356(88)90138-2

Kelloway, E. K., Gottlieb, B. H., & Barham, L. (1999). The source, nature, and direction of work and family conflict: A longitudinal investigation. *Journal of Occupational Health Psychology*, *4*(4), 337–346. https://doi.org/10.1037//1076-8998.4.4.337

Kopelman, R. E., Greenhaus, J. H., & Connolly, T. F. (1983). A model of work, family, and interrole conflict: A construct validation study. *Organizational Behavior and Human Performance*, *32*(2), 198–215. https://doi.org/10.1016/0030-5073(83)90147-2

Kuntsche, S., & Kuntsche, E. (2021). When the Burden Gets Overwhelming: Testing an Inverse U-Shaped Relation between Work-Family Conflicts and Alcohol Use. *EUROPEAN ADDICTION RESEARCH*, *27*(1), 42–48. https://doi.org/10.1159/000507665

Lallukka, T., Ferrie, J. E., Kivimaki, M., Shipley, M. J., Sekine, M., Tatsuse, T., Pietilainen, O., Rahkonen, O., Marmot, M. G., & Lahelma, E. (2014). Conflicts Between Work and Family Life and Subsequent Sleep Problems Among Employees from Finland, Britain, and Japan. *INTERNATIONAL JOURNAL OF BEHAVIORAL MEDICINE*, *21*(2), 310–318. https://doi.org/10.1007/s12529-013-9301-6

Lee, S., Crain, T. L., McHale, S. M., Almeida, D. M., & Buxton, O. M. (2017). Daily antecedents and consequences of nightly sleep. *JOURNAL OF SLEEP RESEARCH*, *26*(4), 498–509. https://doi.org/10.1111/jsr.12488

Lee, S.-B., Park, H.-J., & Yoon, M.-S. (2022). A longitudinal analysis on determinants of problem drinking among Korean women: Focusing on a gender perspective. *SUBSTANCE ABUSE TREATMENT PREVENTION AND POLICY*, *17*(1), 52. https://doi.org/10.1186/s13011-022-00481-3

Leineweber, C., Baltzer, M., Magnusson Hanson, L. L., & Westerlund, H. (2013). Work-family conflict and health in Swedish working women and men: A 2-year prospective analysis (the SLOSH study). *European Journal of Public Health*, *23*(4), 710–716. https://doi.org/10.1093/eurpub/cks064

Lopez, C., Sanchez, M. D., Ponte, L., & Ojeda, L. (2022). Work-family interface on hazardous alcohol use and increased risk for prescription drug misuse among diverse working parents in STEM. *AMERICAN JOURNAL OF DRUG AND ALCOHOL ABUSE*, *48*(1), 78–87. https://doi.org/10.1080/00952990.2021.1992771

Macy, J. T., Chassin, L., & Presson, C. C. (2013). Association Between Work-Family Conflict and Smoking Quantity Among Daily Smokers. *NICOTINE & TOBACCO RESEARCH*, *15*(11), 1867–1872. https://doi.org/10.1093/ntr/ntt071

Magee, C. A., Robinson, L. D., & McGregor, A. (2018). The Work-Family Interface and Sleep Quality. *BEHAVIORAL SLEEP MEDICINE*, *16*(6), 601–610. https://doi.org/10.1080/15402002.2016.1266487

Makela, L., Bergbom, B., Tanskanen, J., & Kinnunen, U. (2014). The relationship between international business travel and sleep problems via work-family conflict. *CAREER DEVELOPMENT INTERNATIONAL*, *19*(7), 794–812. https://doi.org/10.1108/CDI-04-2014-0048

Marshall, N. L., & Barnett, R. C. (1993). Work-family strains and gains among two-earner couples. *Journal of Community Psychology*, *21*(1), 64–78. https://doi.org/10.1002/1520-6629(199301)21:1<64::AID-JCOP2290210108>3.0.CO;2-P

Mayfield, D., McLeod, G., & Hall, P. (1974). The CAGE questionnaire: Validation of a new alcoholism screening instrument. *The American Journal of Psychiatry*, *131*(10), 1121–1123. https://doi.org/10.1176/ajp.131.10.1121

Mohr, C. D., Armeli, S., Tennen, H., Temple, M., Todd, M., Clark, J., & Carney, M. A. (2005). Moving beyond the keg party: A daily process study of college student drinking motivations. *Psychology of Addictive Behaviors: Journal of the Society of Psychologists in Addictive Behaviors*, *19*(4), 392–403. https://doi.org/10.1037/0893-164X.19.4.392

Mojza, E. J., Lorenz, C., Sonnentag, S., & Binnewies, C. (2010). Daily recovery experiences: The role of volunteer work during leisure time. *Journal of Occupational Health Psychology*, *15*(1), 60–74. https://doi.org/10.1037/a0017983

Nelson, C. C., Li, Y., Sorensen, G., & Berkman, L. F. (2012). Assessing the Relationship Between Work-Family Conflict and Smoking. *AMERICAN JOURNAL OF PUBLIC HEALTH*, *102*(9), 1767–1772. https://doi.org/10.2105/AJPH.2011.300413

Netemeyer, R. G., Boles, J. S., & McMurrian, R. (1996). Development and validation of work–family conflict and family–work conflict scales. *Journal of Applied Psychology*, *81*(4), 400–410. https://doi.org/10.1037/0021-9010.81.4.400

Osaki, Y., Ino, A., Matsushita, S., Higuchi, S., Kondo, Y., & Kinjo, A. (2014). Reliability and validity of the alcohol use disorders identification test—Consumption in screening for adults with alcohol use disorders and risky drinking in Japan. *Asian Pacific Journal of Cancer Prevention: APJCP*, *15*(16), 6571–6574. https://doi.org/10.7314/apjcp.2014.15.16.6571

Oshio, T., Inoue, A., & Tsutsumi, A. (2017). Does work-to-family conflict really matter for health? Cross-sectional, prospective cohort and fixed-effects analyses. *Social Science & Medicine (1982)*, *175*, 36–42. https://doi.org/10.1016/j.socscimed.2016.12.039

Pagnan, C. E., Seidel, A., & Wadsworth, S. M. (2017). I Just Can't Fit It in! Implications of the Fit Between Work and Family on Health-Promoting Behaviors. *JOURNAL OF FAMILY ISSUES*, *38*(11), 1577–1603. https://doi.org/10.1177/0192513X16631016

Park, H. I., Jang, J., & Nam, J. S. (2023). Physical activity buffers the effects of work-family conflict on work engagement through mastery recovery experience. *CURRENT PSYCHOLOGY*, *42*(1), 348–358. https://doi.org/10.1007/s12144-021-01463-7

Partinen, M., & Gislason, T. (1995). Basic Nordic Sleep Questionnaire (BNSQ): A quantitated measure of subjective sleep complaints. *Journal of Sleep Research*, *4*(S1), 150–155. https://doi.org/10.1111/j.1365-2869.1995.tb00205.x

Pasman, L., & Thompson, J. K. (1988). Body image and eating disturbance in obligatory runners, obligatory weightlifters, and sedentary individuals. *International Journal of Eating Disorders*, *7*(6), 759–769. https://doi.org/10.1002/1098-108X(198811)7:6<759::AID-EAT2260070605>3.0.CO;2-G

Patterson, C. R., Bennett, J. B., & Wiitala, W. L. (2005). Healthy and Unhealthy Stress Unwinding: Promoting Health in Small Businesses. *Journal of Business and Psychology*, *20*(2), 221–247. https://doi.org/10.1007/s10869-005-8261-5

Pitsopoulos, C. N., & and Greenwood, K. M. (2002). Problems with the measures of gastrointestinal and cardiovascular symptom frequency in The Standard Shiftwork Index. *Work & Stress*, *16*(1), 70–78. https://doi.org/10.1080/02678370110060928

Roberto, K. J., & Taylor, J. F. (2022). Alcohol and cigarette use affecting the relationship between work-life conflict and physical health. *COMMUNITY WORK & FAMILY*, *25*(2), 232–239. https://doi.org/10.1080/13668803.2020.1740171

Schat, A. C. H., Kelloway, E. K., & Desmarais, S. (2005). The Physical Health Questionnaire (PHQ): Construct validation of a self-report scale of somatic symptoms. *Journal of Occupational Health Psychology*, *10*(4), 363–381. https://doi.org/10.1037/1076-8998.10.4.363

Scholarios, D., Hesselgreaves, H., & Pratt, R. (2017). Unpredictable working time, well-being and health in the police service. *INTERNATIONAL JOURNAL OF HUMAN RESOURCE MANAGEMENT*, *28*(16), 2275–2298. https://doi.org/10.1080/09585192.2017.1314314

Sekine, M., Chandola, T., Martikainen, P., Marmot, M., & Kagamimori, S. (2006). Work and family characteristics as determinants of socioeconomic and sex inequalities in sleep: The Japanese civil servants study. *SLEEP*, *29*(2), 206–216. https://doi.org/10.1093/sleep/29.2.206

Shin, D. S., & Jeong, B. Y. (2020). Relationship between Negative Work Situation, Work-Family Conflict, Sleep-Related Problems, and Job Dissatisfaction in the Truck Drivers. *SUSTAINABILITY*, *12*(19), 8114. https://doi.org/10.3390/su12198114

Shukri, M., Jones, F., & Conner, M. (2016). Work Factors, Work-Family Conflict, the Theory of Planned Behaviour and Healthy Intentions: A Cross-Cultural Study. *Stress and Health: Journal of the International Society for the Investigation of Stress*, *32*(5), 559–568. https://doi.org/10.1002/smi.2662

Shukri, M., Jones, F., & Conner, M. (2018). Relationship between work-family conflict and unhealthy eating: Does eating style matter? *Appetite*, *123*, 225–232. https://doi.org/10.1016/j.appet.2017.12.027

Slopen, N., Kontos, E. Z., Ryff, C. D., Ayanian, J. Z., Albert, M. A., & Williams, D. R. (2013). Psychosocial stress and cigarette smoking persistence, cessation, and relapse over 9-10 years: A prospective study of middle-aged adults in the United States. *CANCER CAUSES & CONTROL*, *24*(10), 1849–1863. https://doi.org/10.1007/s10552-013-0262-5

Vleeshouwers, J., Knardahl, S., & Christensen, J. O. (2019). A prospective study of work-private life conflict and number of pain sites: Moderated mediation by sleep problems and support. *JOURNAL OF BEHAVIORAL MEDICINE*, *42*(2), 234–245. https://doi.org/10.1007/s10865-018-9957-0

Wang, M., Liu, S., Zhan, Y., & Shi, J. (2010). Daily Work-Family Conflict and Alcohol Use: Testing the Cross-Level Moderation Effects of Peer Drinking Norms and Social Support. *JOURNAL OF APPLIED PSYCHOLOGY*, *95*(2), 377–386. https://doi.org/10.1037/a0018138

Wei, H., Wu, B., Park, H., & Deng, C. (2023). Is exercise good for all? Time- and strain-based work-family conflict and its impacts. *The Journal of Social Psychology*, *163*(2), 230–247. https://doi.org/10.1080/00224545.2022.2111251

Wilsnack, S. C., Klassen, A. D., Schur, B. E., & Wilsnack, R. W. (1991). Predicting onset and chronicity of women's problem drinking: A five-year longitudinal analysis. *American Journal of Public Health*, *81*(3), 305–318. https://doi.org/10.2105/ajph.81.3.305

Windle, M. (2020). Maturing Out of Alcohol Use in Young Adulthood: Latent Class Growth Trajectories and Concurrent Young Adult Correlates. *Alcoholism, Clinical and Experimental Research*, *44*(2), 532–540. https://doi.org/10.1111/acer.14268

Wolff, J. M., Rospenda, K. M., Richman, J. A., Liu, L., & Milner, L. A. (2013). Work-family conflict and alcohol use: Examination of a moderated mediation model. *Journal of Addictive Diseases*, *32*(1), 85–98. https://doi.org/10.1080/10550887.2012.759856

Yu, L., Buysse, D. J., Germain, A., Moul, D. E., Stover, A., Dodds, N. E., Johnston, K. L., & Pilkonis, P. A. (2011). Development of short forms from the PROMIS™ sleep disturbance and Sleep-Related Impairment item banks. *Behavioral Sleep Medicine*, *10*(1), 6–24. https://doi.org/10.1080/15402002.2012.636266

Zhang, Y., Duffy, J. F., & De Castillero, E. R. (2017). Do sleep disturbances mediate the association between work-family conflict and depressive symptoms among nurses? A cross-sectional study. *Journal of Psychiatric and Mental Health Nursing*, *24*(8), 620–628. https://doi.org/10.1111/jpm.12409

Table S4. Risk of bias assessment for the included studies.

Study (Year)	1.Question / objective sufficiently described?	2.Study design evident and appropriate?	3.Method of subject/comparison group selection or source of information/input variables described and appropriate?	4.Subject (and comparison group, if applicable) characteristics sufficiently described?	5.If interventional and random allocation was possible, was it described?	6.If interventional and blinding of investigators was possible, was it reported?	7.If interventional and blinding of subjects was possible, was it reported?	8.Outcome and (if applicable) exposure measure(s) well defined and robust to measurement /misclassification bias? Means of assessment reported?	9.Sample size appropriate?	10.Analytic methods described/justified and appropriate?	11.Some estimate of variance is reported for the main results?	12.Controlled for confounding?	13.Results reported in sufficient detail?	14.Conclusions supported by the results?	Total
(Zhang et al., 2017)	2	2	1	2	NA	NA	NA	2	2	2	2	2	2	2	21
(Oshio et al., 2017)	2	2	2	2	NA	NA	NA	2	2	2	2	2	2	2	22
(Kuntsche & Kuntsche, 2021)	2	2	1	2	NA	NA	NA	2	2	2	2	2	2	2	21

Study (Year)	1.Question / objective sufficiently described?	2.Study design evident and appropriate?	3.Method of subject/comparison group selection or source of information/input variables described and appropriate?	4.Subject (and comparison group, if applicable) characteristics sufficiently described?	5.If interventional and random allocation was possible, was it described?	6.If interventional and blinding of investigators was possible, was it reported?	7.If interventional and blinding of subjects was possible, was it reported?	8.Outcome and (if applicable) exposure measure(s) well defined and robust to measurement /misclassification bias? Means of assessment reported?	9.Sample size appropriate?	10.Analytic methods described/justified and appropriate?	11.Some estimate of variance is reported for the main results?	12.Controlled for confounding?	13.Results reported in sufficient detail?	14.Conclusions supported by the results?	Total
(Shukri et al., 2018)	2	2	1	2	NA	NA	NA	2	2	2	0	2	2	2	19
(Allen & Armstrong, 2006)	2	2	2	2	NA	NA	NA	2	2	2	0	2	2	2	20
(Magee et al., 2018)	2	2	2	2	NA	NA	NA	2	2	2	2	2	2	2	22
(Makela et al., 2014)	2	2	2	2	NA	NA	NA	2	2	2	0	2	2	2	20

Study (Year)	1.Question / objective sufficiently described?	2.Study design evident and appropriate?	3.Method of subject/comparison group selection or source of information/input variables described and appropriate?	4.Subject (and comparison group, if applicable) characteristics sufficiently described?	5.If interventional and random allocation was possible, was it described?	6.If interventional and blinding of investigators was possible, was it reported?	7.If interventional and blinding of subjects was possible, was it reported?	8.Outcome and (if applicable) exposure measure(s) well defined and robust to measurement /misclassification bias? Means of assessment reported?	9.Sample size appropriate?	10.Analytic methods described/justified and appropriate?	11.Some estimate of variance is reported for the main results?	12.Controlled for confounding?	13.Results reported in sufficient detail?	14.Conclusions supported by the results?	Total
(Eshak, 2019)	2	2	2	2	NA	NA	NA	2	2	2	2	2	2	2	22
(Aazami et al., 2016)	2	2	2	2	NA	NA	NA	2	2	2	1	2	2	2	21
(Shin & Jeong, 2020)	2	2	2	2	NA	NA	NA	2	2	2	2	0	2	2	20
(Vleeshouwers et al., 2019)	2	2	1	2	NA	NA	NA	2	2	2	2	2	2	2	21

Study (Year)	1.Question / objective sufficiently described?	2.Study design evident and appropriate?	3.Method of subject/comparison group selection or source of information/input variables described and appropriate?	4.Subject (and comparison group, if applicable) characteristics sufficiently described?	5.If interventional and random allocation was possible, was it described?	6.If interventional and blinding of investigators was possible, was it reported?	7.If interventional and blinding of subjects was possible, was it reported?	8.Outcome and (if applicable) exposure measure(s) well defined and robust to measurement /misclassification bias? Means of assessment reported?	9.Sample size appropriate?	10.Analytic methods described/justified and appropriate?	11.Some estimate of variance is reported for the main results?	12.Controlled for confounding?	13.Results reported in sufficient detail?	14.Conclusions supported by the results?	Total
(Lallukka et al., 2014)	2	2	2	2	NA	NA	NA	2	2	2	2	2	2	2	22
(Scholarios et al., 2017)	2	2	2	2	NA	NA	NA	2	2	2	2	2	2	2	22
(Crain et al., 2014)	2	2	2	2	NA	NA	NA	2	2	2	2	2	2	2	22
(Sekine et al., 2006)	2	2	2	2	NA	NA	NA	2	2	2	2	2	2	2	22

Study (Year)	1.Question / objective sufficiently described?	2.Study design evident and appropriate?	3.Method of subject/comparison group selection or source of information/input variables described and appropriate?	4.Subject (and comparison group, if applicable) characteristics sufficiently described?	5.If interventional and random allocation was possible, was it described?	6.If interventional and blinding of investigators was possible, was it reported?	7.If interventional and blinding of subjects was possible, was it reported?	8.Outcome and (if applicable) exposure measure(s) well defined and robust to measurement /misclassification bias? Means of assessment reported?	9.Sample size appropriate?	10.Analytic methods described/justified and appropriate?	11.Some estimate of variance is reported for the main results?	12.Controlled for confounding?	13.Results reported in sufficient detail?	14.Conclusions supported by the results?	Total
(S. Lee et al., 2017)	2	2	2	2	NA	NA	NA	2	2	2	2	2	2	2	22
(Lopez et al., 2022)	2	2	2	2	NA	NA	NA	2	2	2	1	2	2	2	21
(Roberto & Taylor, 2022)	2	2	1	2	NA	NA	NA	2	2	1	0	2	2	2	18
(Wang et al., 2010)	2	2	1	2	NA	NA	NA	2	2	2	2	2	2	2	21

Study (Year)	1.Question / objective sufficiently described?	2.Study design evident and appropriate?	3.Method of subject/comparison group selection or source of information/input variables described and appropriate?	4.Subject (and comparison group, if applicable) characteristics sufficiently described?	5.If interventional and random allocation was possible, was it described?	6.If interventional and blinding of investigators was possible, was it reported?	7.If interventional and blinding of subjects was possible, was it reported?	8.Outcome and (if applicable) exposure measure(s) well defined and robust to measurement /misclassification bias? Means of assessment reported?	9.Sample size appropriate?	10.Analytic methods described/justified and appropriate?	11.Some estimate of variance is reported for the main results?	12.Controlled for confounding?	13.Results reported in sufficient detail?	14.Conclusions supported by the results?	Total
(S.-B. Lee et al., 2022)	2	2	2	2	NA	NA	NA	2	2	2	2	2	2	2	22
(Windle, 2020)	2	2	1	2	NA	NA	NA	2	2	2	0	1	2	2	18
(Wolff et al., 2013)	2	2	2	2	NA	NA	NA	2	2	2	2	2	2	2	22
(Leineweber et al., 2013)	2	2	2	2	NA	NA	NA	2	2	2	2	2	2	2	22
(Bennett et	2	2	1	2	NA	NA	NA	2	2	2	0	2	2	2	19

Study (Year)	1.Question / objective sufficiently described?	2.Study design evident and appropriate?	3.Method of subject/comparison group selection or source of information/input variables described and appropriate?	4.Subject (and comparison group, if applicable) characteristics sufficiently described?	5.If interventional and random allocation was possible, was it described?	6.If interventional and blinding of investigators was possible, was it reported?	7.If interventional and blinding of subjects was possible, was it reported?	8.Outcome and (if applicable) exposure measure(s) well defined and robust to measurement /misclassification bias? Means of assessment reported?	9.Sample size appropriate?	10.Analytic methods described/justified and appropriate?	11.Some estimate of variance is reported for the main results?	12.Controlled for confounding?	13.Results reported in sufficient detail?	14.Conclusions supported by the results?	Total
al., 2006)															
(Nelson et al., 2012)	2	2	1	2	NA	NA	NA	2	2	2	2	2	2	2	21
(Macy et al., 2013)	2	2	2	2	NA	NA	NA	2	2	2	2	0	2	2	20
(Slopen et al., 2013)	2	2	2	2	NA	NA	NA	2	2	2	2	2	2	2	22
(Park et al., 2023)	2	2	2	2	NA	NA	NA	2	2	2	2	0	2	2	20

Study (Year)	1.Question / objective sufficiently described?	2.Study design evident and appropriate?	3.Method of subject/comparison group selection or source of information/input variables described and appropriate?	4.Subject (and comparison group, if applicable) characteristics sufficiently described?	5.If interventional and random allocation was possible, was it described?	6.If interventional and blinding of investigators was possible, was it reported?	7.If interventional and blinding of subjects was possible, was it reported?	8.Outcome and (if applicable) exposure measure(s) well defined and robust to measurement /misclassification bias? Means of assessment reported?	9.Sample size appropriate?	10.Analytic methods described/justified and appropriate?	11.Some estimate of variance is reported for the main results?	12.Controlled for confounding?	13.Results reported in sufficient detail?	14.Conclusions supported by the results?	Total
(Pagnan et al., 2017)	2	2	2	2	NA	NA	NA	2	2	2	0	2	2	2	20
(Clayton et al., 2015)	2	2	2	2	NA	NA	NA	2	2	2	0	2	2	2	20
(Wei et al., 2023)	2	2	1	2	NA	NA	NA	2	2	2	2	2	2	2	21
(Shukri et al., 2016)	2	2	1	2	NA	NA	NA	2	2	2	1	0	2	2	18

Study (Year)	1.Question / objective sufficiently described?	2.Study design evident and appropriate?	3.Method of subject/comparison group selection or source of information/input variables described and appropriate?	4.Subject (and comparison group, if applicable) characteristics sufficiently described?	5.If interventional and random allocation was possible, was it described?	6.If interventional and blinding of investigators was possible, was it reported?	7.If interventional and blinding of subjects was possible, was it reported?	8.Outcome and (if applicable) exposure measure(s) well defined and robust to measurement /misclassification bias? Means of assessment reported?	9.Sample size appropriate?	10.Analytic methods described/justified and appropriate?	11.Some estimate of variance is reported for the main results?	12.Controlled for confounding?	13.Results reported in sufficient detail?	14.Conclusions supported by the results?	Total
(Guérin & Gottschall, 2024)	2	2	1	2	NA	NA	NA	2	2	2	2	1	2	2	20

Table S5. The relationship between Work-family conflict and health behaviors in included studies.

Study (Year)	Types of work-life balance	Types of health behaviors	Relationship
(Zhang et al., 2017)	Work-family conflict	Sleep disturbances	Work-family conflict positively and significantly associated with sleep disturbances.
(Oshio et al., 2017)	Work-family conflict	Current smoking, problem drinking, leisure-time physical inactivity, sickness absence, and refraining from medical care	Work-family conflict was significantly and positively associated with current smoking, problem drinking, leisure-time physical inactivity, sickness absence, and refraining from medical care.
(Kuntsche & Kuntsche, 2021)	Work-family conflict	Alcohol use	with low and high level of conflict report drinking less alcohol than those with a medium level of conflict.
(Shukri et al., 2018)	Work-family conflict	Unhealthy eating	There was significant relationship between Work-family conflict and unhealthy eating.
(Allen & Armstrong, 2006)	Work-family conflict	Physical activity, fatty food consumption, and healthy food consumption	Work-family conflict was associated with less physical activity and with eating more high fat foods; WIF was associated with eating fewer healthy foods.
(Magee et al., 2018)	Work-family conflict, Work-family enrichment	Sleep quality	Work-family conflict was associated with poorer sleep quality. Work-family enrichment was not directly associated with sleep quality, but was indirectly associated with better sleep quality.
(Makela et al., 2014)	Work-family conflict	Sleep problem	Work-family conflict was positively and significantly associated with subsequent sleep problems.
(Eshak, 2019)	Work-family conflict	Sleep disorders	Work-family conflict positively and significantly associated with sleep disorders.
(Aazami et al., 2016)	Work-family conflict	Sleep disturbances	high level of work-family conflicts significantly increase sleep disturbances

Study (Year)	Types of work-life balance	Types of health behaviors	Relationship
(Shin & Jeong, 2020)	Work-family conflict	Sleep problem	Work-family conflict had significantly affected sleep-related problems
(Vleeshouwers et al., 2019)	Work-private life conflict	Sleep problem	Work-life conflict was positively correlated with sleep problem.
(Lallukka et al., 2014)	Work-family conflict	Sleep problem	Work-family conflict predicted subsequent sleep problems.
(Scholarios et al., 2017)	Work-family conflict	Alcohol consumption, sleep disturbance	Work-family conflict positively and significantly associated with sleep disturbance and alcohol consumption.
(Crain et al., 2014)	Work-family conflict	Sleep quality and quantity	Work-family conflict was significantly related to both objective and self-report measures of sleep quantity and quality
(Sekine et al., 2006)	Work-family conflict	Sleep quality	higher Work-family conflict was independently associated with poorer sleep quality .
(S. Lee et al., 2017)	Work-family conflict	Sleep quality, sleep hours, sleep latency	sleep hours and sleep quality were associated with next-day consequences of Work-life conflict; more work--family conflict, longer sleep latency
(Lopez et al., 2022)	Work-family conflict	Alcohol use	There was no significant relationship between Work-family conflict and alcohol use.
(Roberto & Taylor, 2022)	Work-family conflict	Alcohol and cigarettes use	Work-family conflict was significantly and positively associated with alcohol and cigarettes use.
(Wang et al., 2010)	Work-family conflict	Alcohol use	daily work-to-family conflict but not family-to-work conflict had a significant within-subject main effect on daily alcohol use.
(S.-B. Lee et al., 2022)	Work-family conflict	Problem drinking	work–family conflict stress is a significant predictor of drinking behaviors.
(Windle, 2020)	Work-family conflict	Alcohol use	There was significant and positive correlation between Work-family conflict and alcohol use.
(Wolff et al., 2013)	Work-family conflict	Alcohol use	Significant and positive correlation existed between Work-family conflict and alcohol use.
(Leineweber et al., 2013)	Work-family conflict	Problem drinking	WFC was significantly related to an increased risk for problem

Study (Year)	Types of work-life balance	Types of health behaviors	Relationship
			drinking among men.Not significant in women.
(Bennett et al., 2006)	Work-life conflict	At-risk drinking	life-to-work conflict uniquely predicted ARD for younger workers.
(Nelson et al., 2012)	Work-family conflict	Current smoking	There was significant and positive correlation between Work-family conflict and current smoking.
(Macy et al., 2013)	Work-family conflict	Smoking quantity	work-to-home conflict had a negative impact on smoking quantity for all participants, and home-to-work conflict was associated with smoking quantity for Women.
(Slopen et al., 2013)	Work-family conflict	Smoking behavior	Work-family conflict did not show a modest association with smoking behaviors.
(Park et al., 2023)	Work-family conflict	Physical activity	Significant and negative correlation existed between Work-family conflict and physical activity.
(Pagnan et al., 2017)	Work-family conflict	Physical activity	There was no significant relationship between Work-family conflict and physical activity.
(Clayton et al., 2015)	Work-family conflict	Physical activity	There was no direct and significant relationship between Work-family conflict and physical activity.
(Wei et al., 2023)	Work-family conflict	Physical activity	There was significant relationship between Work-family conflict and physical activity
(Shukri et al., 2016)	Work-family conflict	Healthy diet, physical activity	There was no direct and significant relationship between Work-family conflict and physical activity/healthy diet.
(Guérin & Gottschall, 2024)	Work-family conflict	Physical activity	There was no direct link between work-family conflict and physical activity

Figure S1-S5. Funnel plot of the correlation between work-family conflict and health behaviors.

Figure S1. Funnel plot of the correlation between work-family conflict and sleep disturbances.

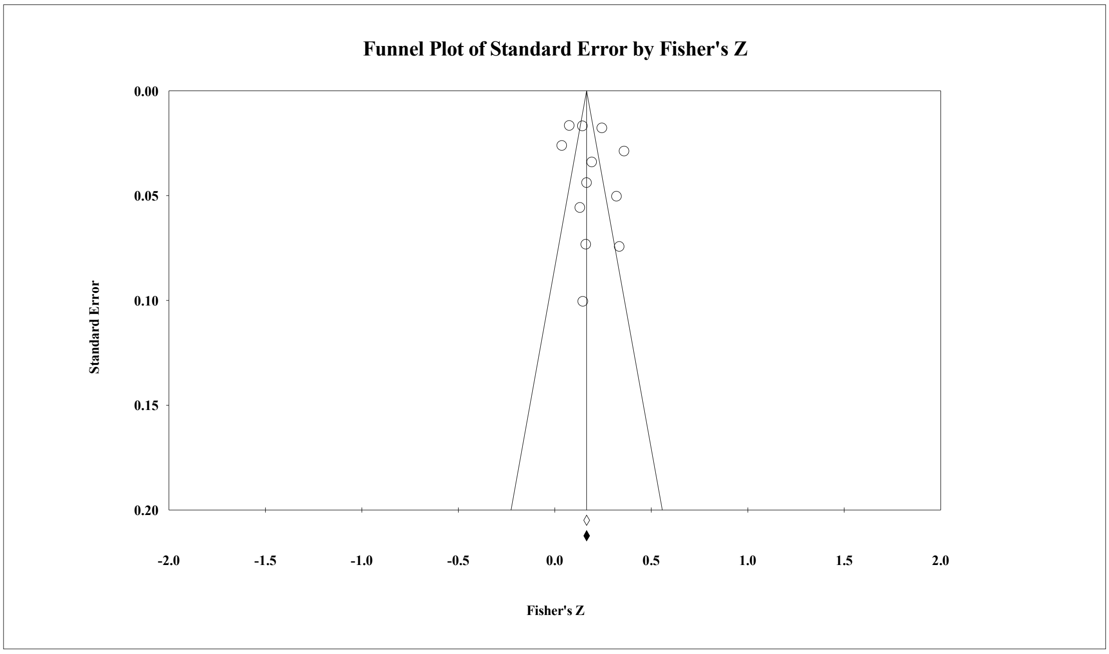

*Egger's Test：P = 0.433

Figure S2. Funnel plot of the correlation between work-family conflict and smoking behaviors

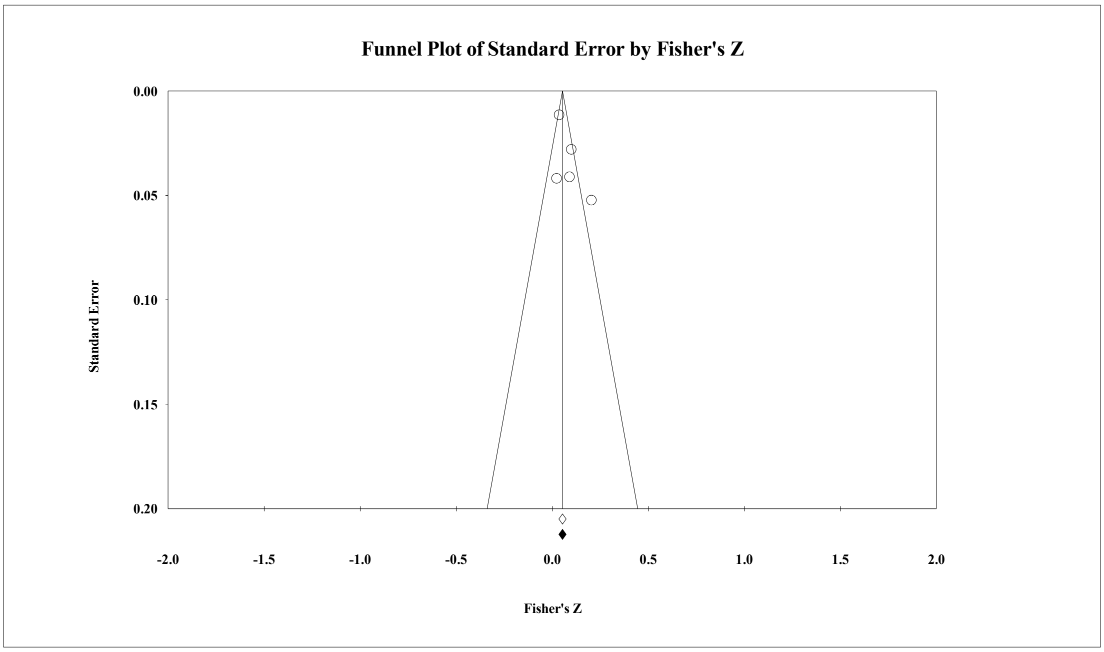

*Egger's Test：P = 0.165

Figure S3. Funnel plot of the correlation between work-family conflict and alcohol consumption.

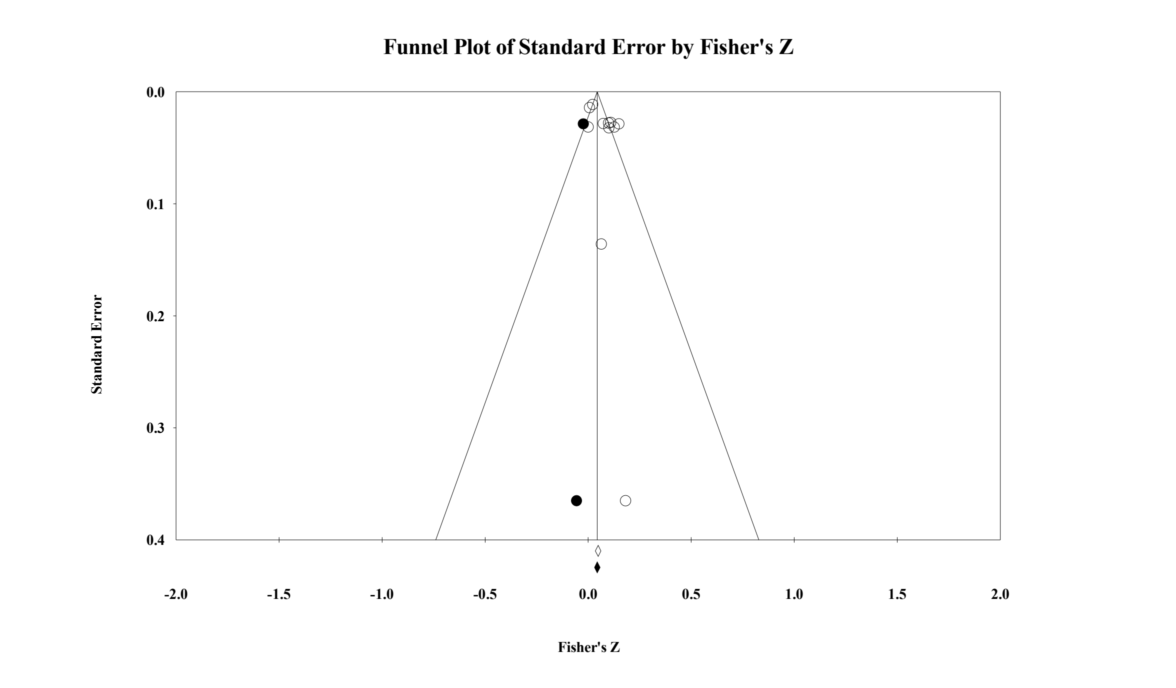

*Egger's Test：P = 0.071

Figure S4. Funnel plot of the correlation between work-family conflict and physical activity levels.

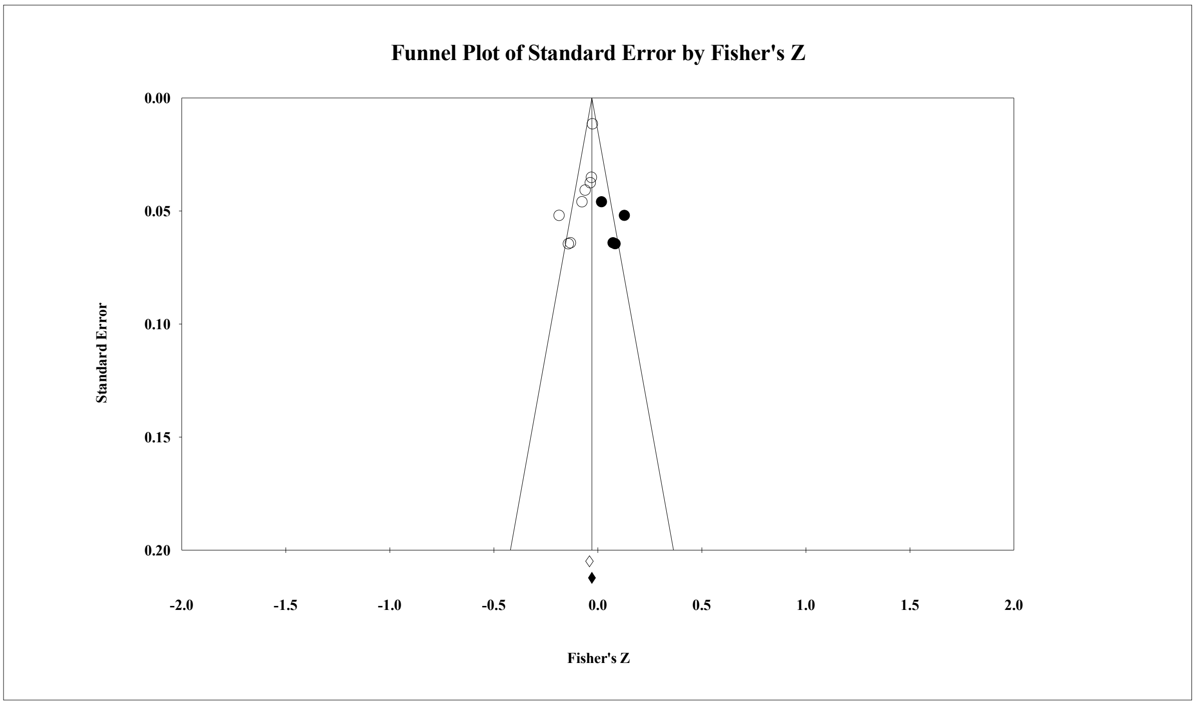

*Egger's Test：P = 0.016

Figure S5. Funnel plot of the correlation between work-family conflict and healthy diet.

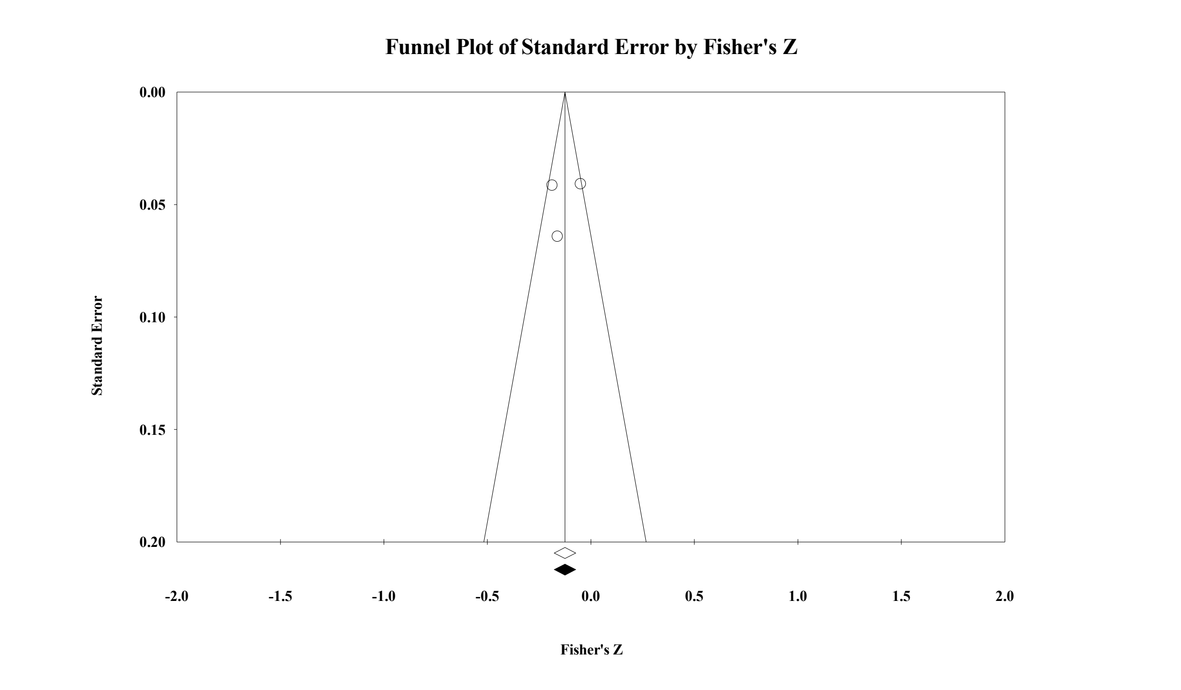

*Egger's Test：P = 0.816
